# Supplementary material for: Covalent organic framework membranes through a mixed-dimensional assembly for molecular separations
Source: Nat Commun. 2019 May 8;10:2101. doi: 10.1038/s41467-019-10157-5 (PMC6506600; doi:10.1038/s41467-019-10157-5)
Supplement: Supplementary file 1 — Supplementary Information [file 41467_2019_10157_MOESM1_ESM.pdf]

Supplementary Information

Covalent organic framework membranes through a mixed-dimensional assembly for molecular separations

Yang et al.

Supplementary methods

Materials

1,3,5-Triformylphloroglucinol (Tp) and triaminoguanidinium chloride (TG_{Cl}) were synthesized according to the literature[1, 2]. The aqueous dispersion of TEMPO-oxidized CNFs (1 wt%, $COO^{-}Na^{+}$ content 1.2 mmol g^{-1}) was produced by softwood pulp according to the literature[3]. Polyacrylonitrile (PAN) substrates with a molecular weight cut-off of 100,000 were bought from Shandong Megavision Membrane Engineering and Technology Co., Ltd (China). All other reagents and solvents were commercially available and used as received.

Synthesis procedures

Synthesis of Tp: Tp was synthesized following the previously reported methods[1]. Briefly, a mixture of hexamethylenetetramine (7.4 g), phloroglucinol (3 g), and trifluoroacetic acid (45 mL) was heated at 100 ˚C for 2.5 h under a nitrogen atmosphere, then 30 mL of 3 M HCl was added to it slowly and the mixture was further refluxed for another 1 h. The mixture was allowed to cool at room temperature and filtered through celite bed. The obtained filtrate was extracted with dichloromethane (4 times × 70 mL) and dried over anhydrous $MgSO_4$. The obtained extract was concentrated by filtration to yield dull yellow colored solid. The obtained crude product was purified by hot ethanol and then dried under vacuum at 120 ^{o}C for 24 h to yield the desired product.

Synthesis of TG_{Cl}. TG_{Cl} was synthesized using hydrazine hydrate in 1,4-dioxane under refluxing condition[2]. Briefly, 1.91 g of guanidine hydrochloride was added to 10 mL of 1,4-dioxane under stirring condition. To it, 3.41 g of hydrazine hydrate was added and the mixture was refluxed for

2 h. Then the mixture was cooled to room temperature, filtered and washed with 1,4-dioxane to remove excess hydrazine hydrate and finally dried to yield TG_{Cl}.

Synthesis of COF $TpTG_{Cl}$: $TpTG_{Cl}$ was synthesized via Schiff-base condensation[2] between 0.2 mmol Tp (42 mg) and 0.2 mmol TG_{Cl} (28 mg) in a sealed Pyrex tube. The mixtures (dioxane: water=2: 0.6 mL) were charged into the Pyrex tube and sonicated for 20 min. The mixtures were degassed under liquid N_2 (77 K) by three freeze-pump-thaw cycles. The Pyrex tube was then vacuum-sealed and kept at room temperature until the reaction mixtures attended the room temperature. The sealed Pyrex tube containing the reaction mixture was kept at 120 ˚C for 3 days. The bulk $TpTG_{Cl}$ was obtained as a brown colored precipitate. The product was washed thoroughly with N,N-dimethylacetamide, water and acetone respectively and dried at 90 ˚C for 24 h under vacuum to obtain the final $TpTG_{Cl}$. The products were then dispersed into deionized water to obtain a suspension of $TpTG_{Cl}$ nanosheets.

Synthesis of COF TpPa-1, TpBD, TpHZ and TpBD(OH)$_2$[4-6]. A Pyrex tube was charged with Tp (0.3 mmol), corresponding diamine [p-phenylenediamine (Pa-1), benzidine (BD), hydrazine hydrate (HZ) and 3,3'-dihydroxybenzidine (BD(OH)$_2$)] (0.45 mmol), 1.5 mL of mesitylene, 1.5 mL of 1,4-dioxane, 0.5 mL of 6 M aqueous acetic acid. The mixture was sonicated for 10 minutes in order to get a homogeneous dispersion. The tube was then flash frozen at 77 K (liquid N_2 bath), degassed by three freeze-pump-thaw cycles, sealed off and then heated at 120 °C for 3 days. The precipitate was collected by filtration, washed with N,N-dimethylacetamide and acetone thrice and then dried at 150 ^{o}C under vacuum for 12 h. The obtained COFs were then ground in a mortar for 45 min with 2 mL of methanol, and then centrifuged by methanol at 8000 rpm. The COF nanosheets were obtained by drying the supernatant.

Exfoliation of the CNFs. The slurry of TEMPO-oxidized cellulose (1 wt%) was under ultrasonic treatment for 2 h, and followed by high-speed centrifugation (10000 rpm, 20 min) to remove the un-exfoliated cellulose fibers. Finally, the resulting CNFs dispersion was diluted to the desired concentration.

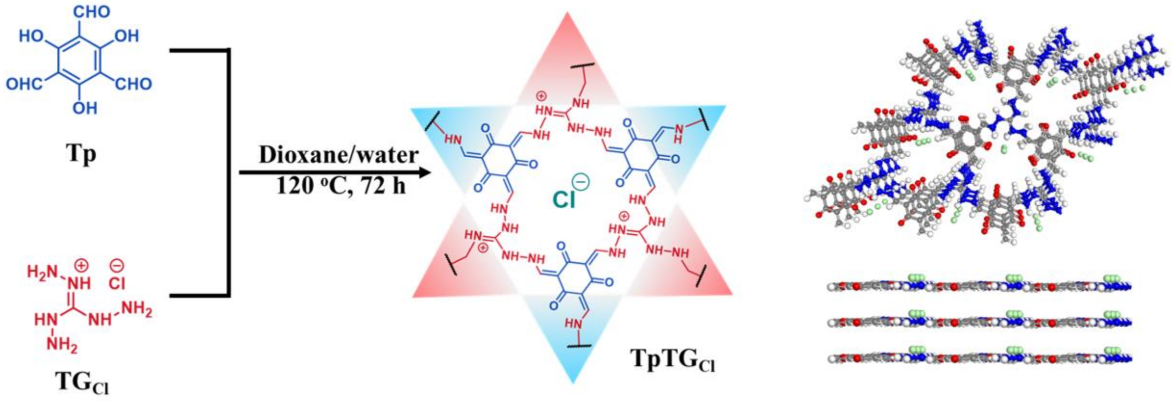

Supplementary Figure 1. A synthetic scheme of $TpTG_{Cl}$ and modeling of $TpTG_{Cl}$ in top view and side view.

Note: The TG_{Cl} with a C_3 symmetric planner structure reacted with C_3 symmetric Tp to form 2D COFs. The irreversible enol to keto tautomerism in COFs occurred. The presence of Cl^- anion sandwiched between two monomer units. Presence of these loosely bound chloride ions and intrinsic positive charge of guanidinium units disturb the *π-π* stacking interactions among the layers in the extended COF structure. Thus, the $TpTG_{Cl}$ possesses a self-exfoliated feature[2].

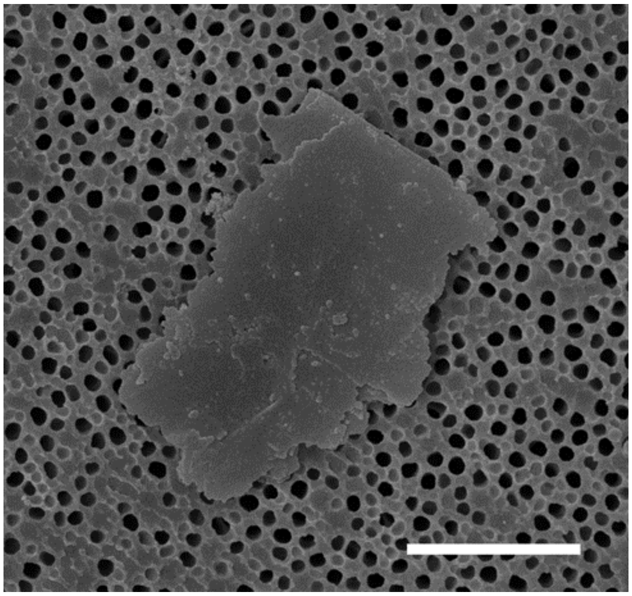

Supplementary Figure 2. SEM surface image of the bulk $TpTG_{Cl}$ on porous anodic aluminum oxide (AAO) (scale bar, 2 μm).

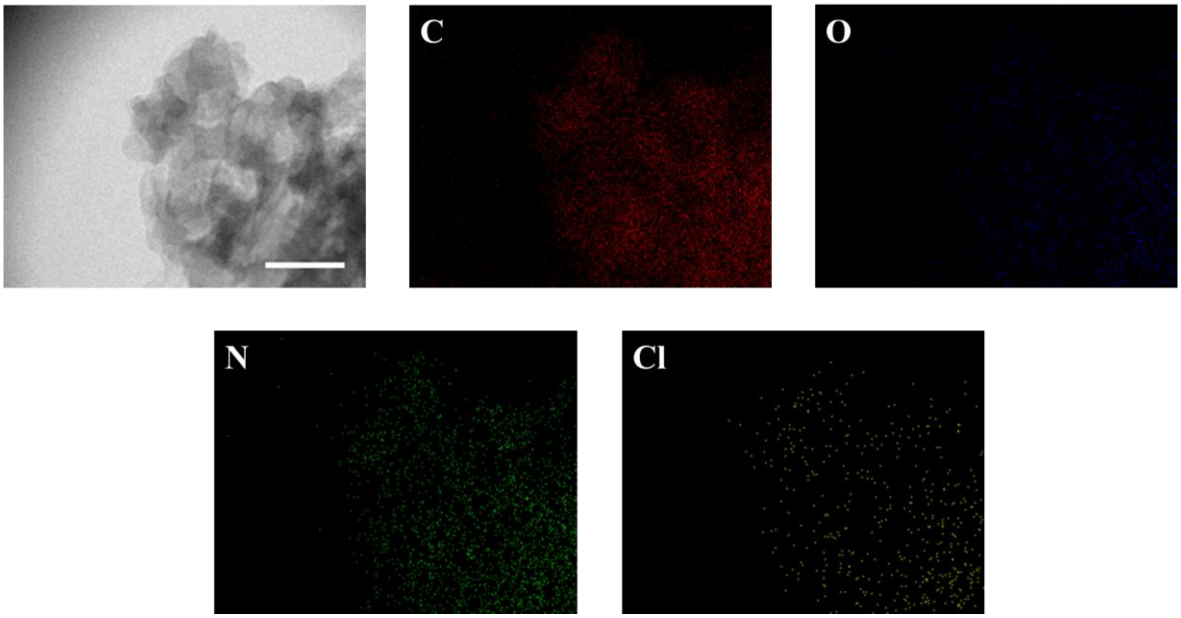

Supplementary Figure 3. TEM image (scale bar, 100 nm) and the corresponding element distribution mappings of the bulk $TpTG_{Cl}$.

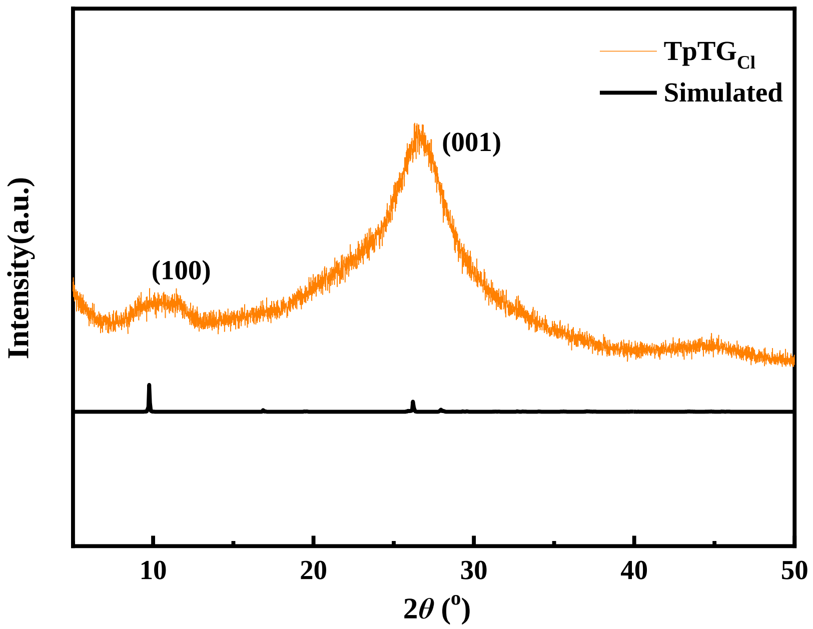

Supplementary Figure 4. PXRD pattern of the TpTG$_{Cl}$.

Note: The first broad peak at $2\theta = \sim 9.7°$ corresponds to (100) plane. The major broad peak at $2\theta = \sim 27.3°$ corresponds to (001) plane, signifying poor π-π stacking between the vertically stacked layers owing to the self-exfoliated structure[2].

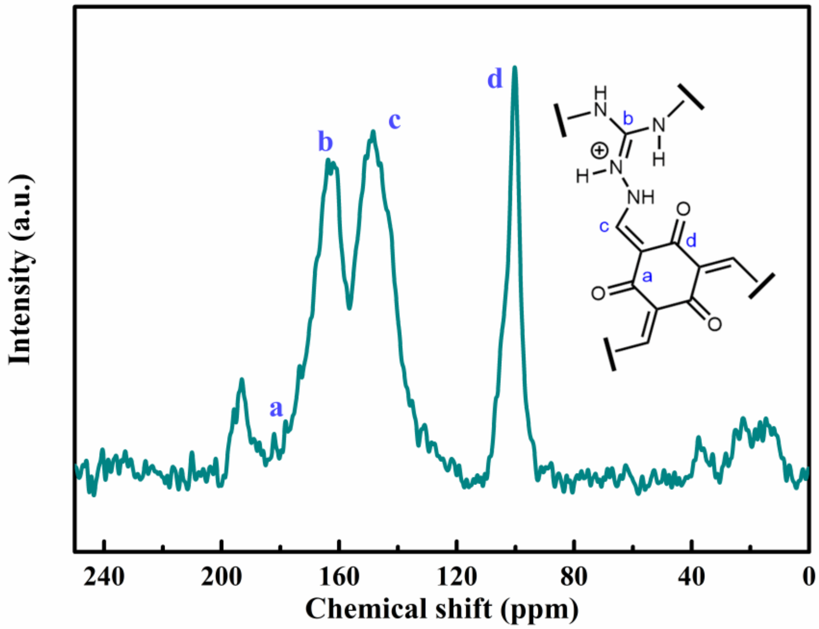

Supplementary Figure 5. ^{13}C CP-MAS solid-state NMR spectrum of the $TpTG_{Cl}$.

Note: The peak at ~100 ppm signified the exocyclic C=C carbon adjacent to the C=O carbon. The C=C carbon attached to the N was obtained at 150 ppm. Carbon signal of guanidinium C=N appeared at ~162 ppm, and a small keto (−C=O) carbon signal was noted in between 180 and 182 ppm. The sharp exocyclic double bond signal is the strong evidence of irreversible enol to keto tautomerism in $TpTG_{Cl}$[2].

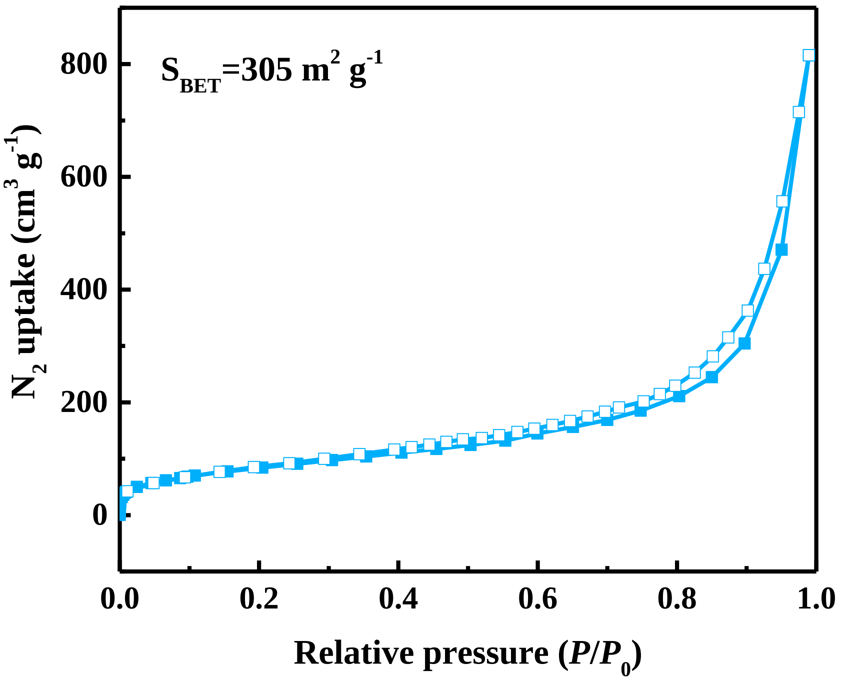

Supplementary Figure 6. N_2 adsorption-desorption isotherms of the $TpTG_{Cl}$.

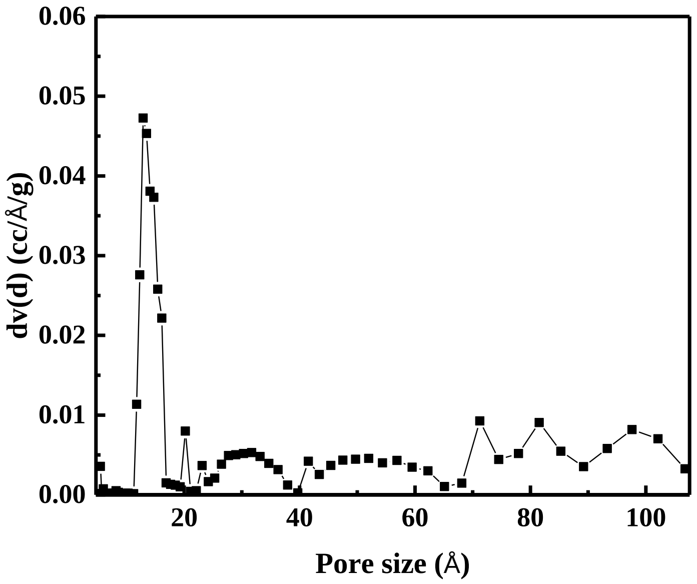

Supplementary Figure 7. Pore size distribution of the $TpTG_{Cl}$.

Supplementary Table 1. The CNF content in $TpTG_{Cl}$@CNFs-X nanocomposites.

Assembled nanocomposites	C/N ratio	CNF content (wt%)
$TpTG_{Cl}$@CNFs-1	0.4476	1.72
$TpTG_{Cl}$@CNFs-2	0.4389	3.49
$TpTG_{Cl}$@CNFs-3	0.4316	5.02
$TpTG_{Cl}$@CNFs-4	0.4236	6.66
$TpTG_{Cl}$@CNFs-5	0.4151	8.42
$TpTG_{Cl}$@CNFs-6	0.4084	9.82
$TpTG_{Cl}$@CNFs-7	0.4015	11.25

Note: The CNF content was calculated by C/N ratio from the element analysis results.

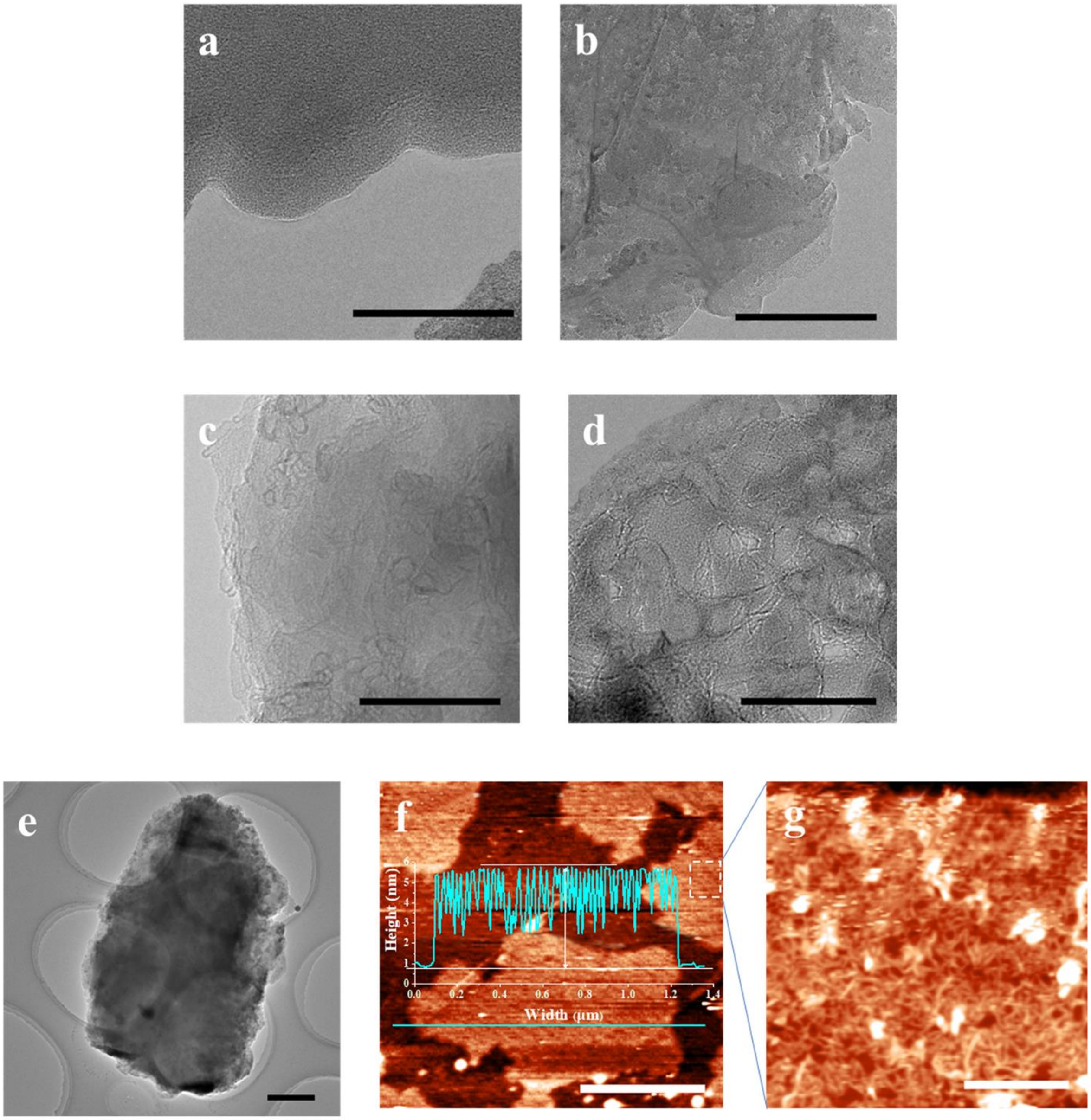

Supplementary Figure 8. High magnification TEM images of a) pure TpTG$_{Cl}$, b) TpTG$_{Cl}$@CNFs-1, c) TpTG$_{Cl}$@CNFs-3 and d) TpTG$_{Cl}$@CNFs-5 (scale bar, 100 nm). Low magnification TEM of e) TpTG$_{Cl}$@CNFs-5 (scale bar, 1 μm). f) AFM image and height profile of TpTG$_{Cl}$@CNFs-5 (scale bar, 500 nm). g) AFM image with a high magnification from the white square in f) (scale bar, 50 nm).

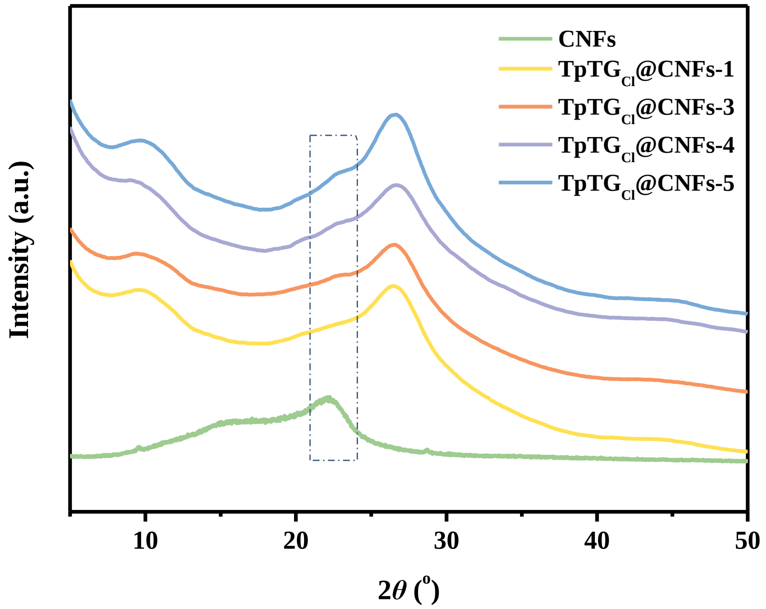

Supplementary Figure 9. PXRD patterns of the CNFs and $TpTG_{Cl}$@CNFs-X nanocomposites.

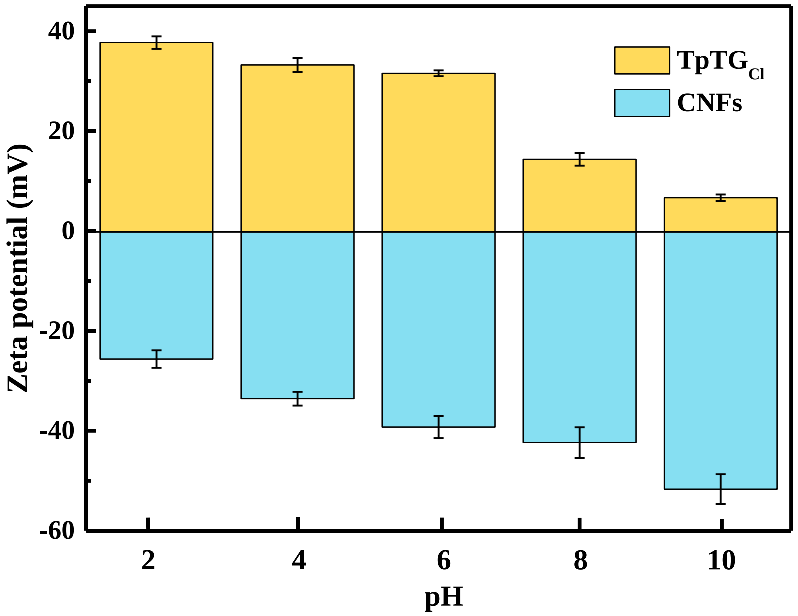

Supplementary Figure 10. Zeta potential of the $TpTG_{Cl}$ aqueous solution and the CNF aqueous solution with pH of 2-10. The concentration of both solutions is 0.1 mg mL^{-1}. Error bars represent standard deviations for 3 measurements.

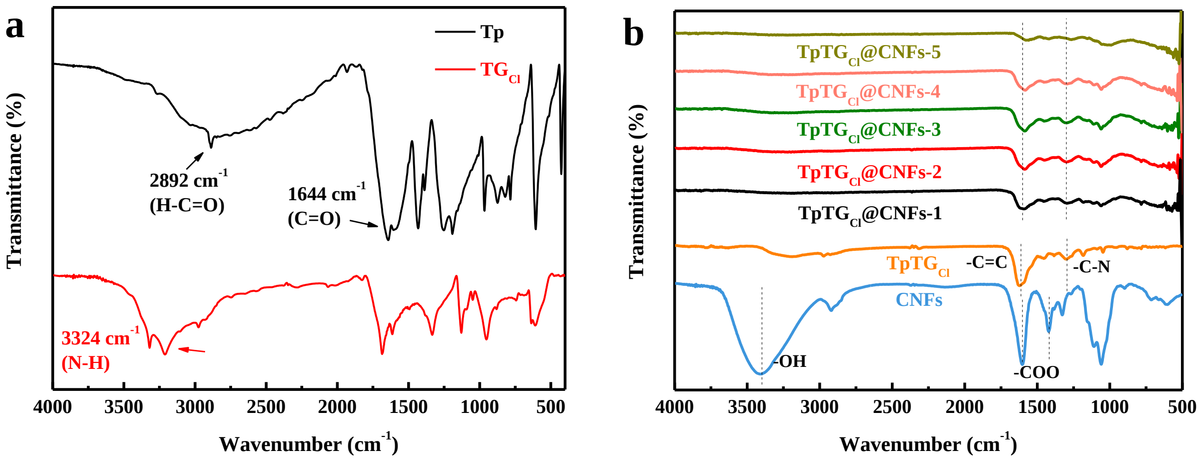

Supplementary Figure 11. FTIR spectra of a) Tp and TG_{Cl}, and b) $TpTG_{Cl}$, CNFs and $TpTG_{Cl}$@CNFs-X nanocomposites.

Note: FTIR spectra reveal the formation of $TpTG_{Cl}$ from corresponding amine (TG_{Cl}) and aldehyde (Tp). Characteristic carbonyl (−C=O) stretching frequency (1644 cm^{-1}), C−H stretching frequency (2892 cm^{-1}) of Tp and N−H stretching frequency (3324 cm^{-1}) of TG_{Cl} are disappeared in the corresponding product. A characteristic C=C stretching frequency at 1597 cm^{-1} and the C−N stretching at 1291 cm^{-1} are observed for $TpTG_{Cl}$[2].

For $TpTG_{Cl}$@CNFs-X composites, the characteristic frequencies at 1597 and 1291 cm^{-1} shift to the lower wavenumbers and the intensity of the adsorption bands declines with increasing CNF content, demonstrating the presence of strong interaction between $TpTG_{Cl}$ and CNFs[7].

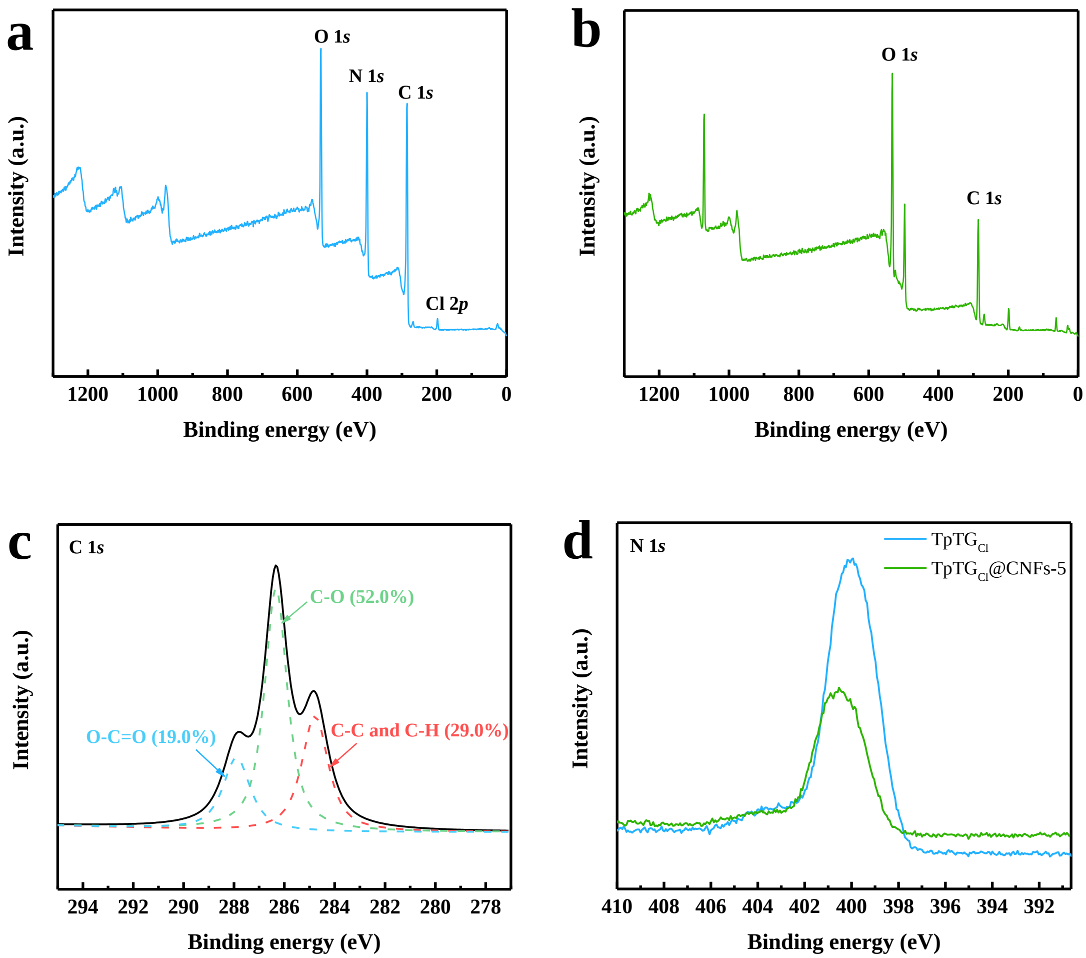

Supplementary Figure 12. Full scan XPS spectra of a) $TpTG_{Cl}$ and b) CNFs. c) High-resolution XPS of C 1*s* peak for CNFs. d) High-resolution XPS of N 1*s* peak for $TpTG_{Cl}$ and $TpTG_{Cl}$@CNFs-5 nanocomposites.

Note: The contents of C−O and O−C=O in CNFs are 52.0% and 19.0%, respectively. The N 1*s* peak of $TpTG_{Cl}$ shifts after assembling with CNFs, indicating the intense interaction between $TpTG_{Cl}$ and CNFs[8].

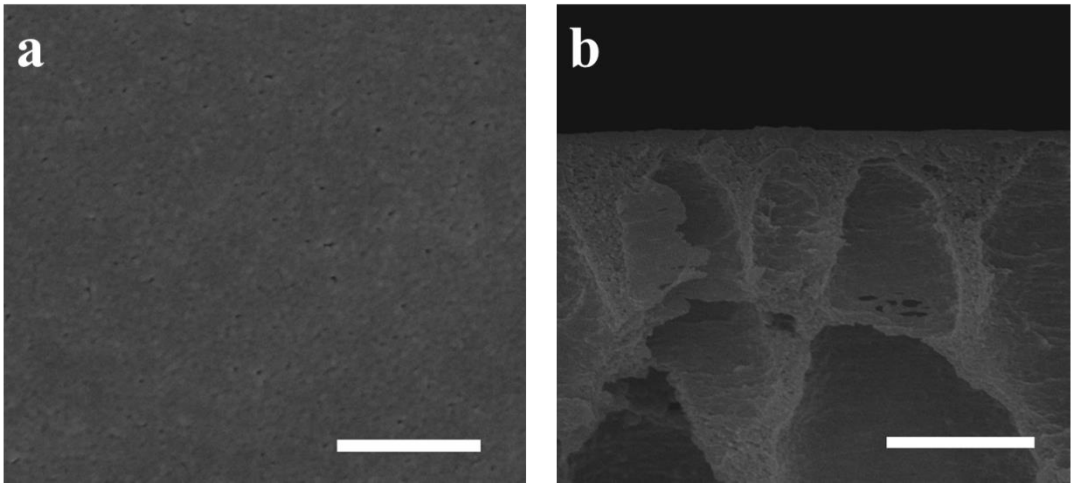

Supplementary Figure 13. a) Surface and b) cross-sectional SEM images of the PAN substrate (scale bar, **a** 500 nm; **b** 2 μm).

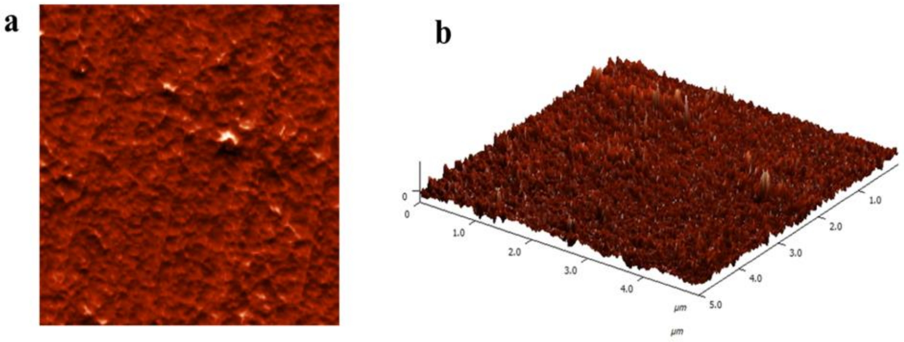

Membrane	Roughness	
	R_a (nm)	R_q (nm)
TpTG$_{Cl}$@CNFs-5/PAN	69.4±3.7	56.3±2.9

Supplementary Figure 14. a) 2D and b) 3D AFM images of the TpTG$_{Cl}$@CNFs/PAN-5 membrane with a scan area of 5 µm × 5 µm, and the corresponding average roughness (R_a) and root-mean-square roughness (R_q). Error bars represent standard deviations for 3 measurements.

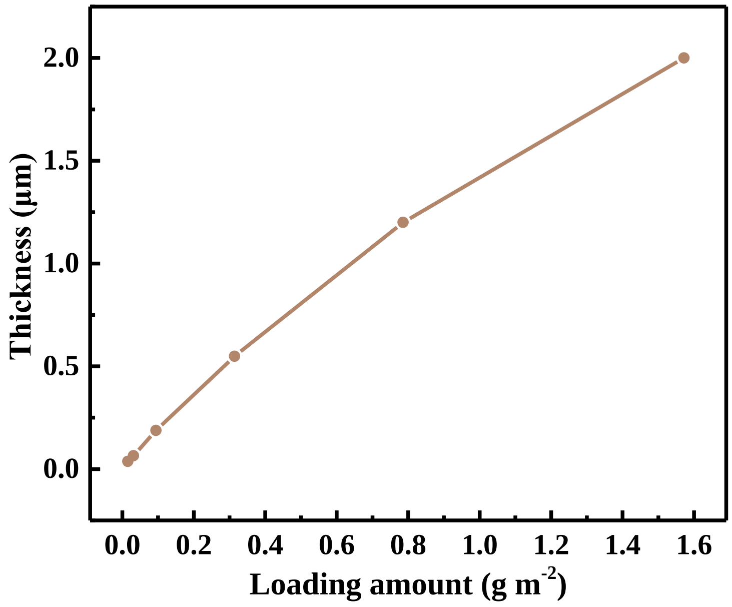

Supplementary Figure 15. Relationship between the thickness of $TpTG_{Cl}$@CNFs-X membrane and the loading amount of filtrated solution. The $TpTG_{Cl}$@CNFs-X solution (0.005 mg mL^{-1}) volume of 5 mL, 10 mL, 30 mL, 100 mL, 250 mL and 500 mL resulted in the membrane thickness of 37 nm, 65 nm, 188 nm, 549 nm, 1.2 μm and 2.0 μm, respectively.

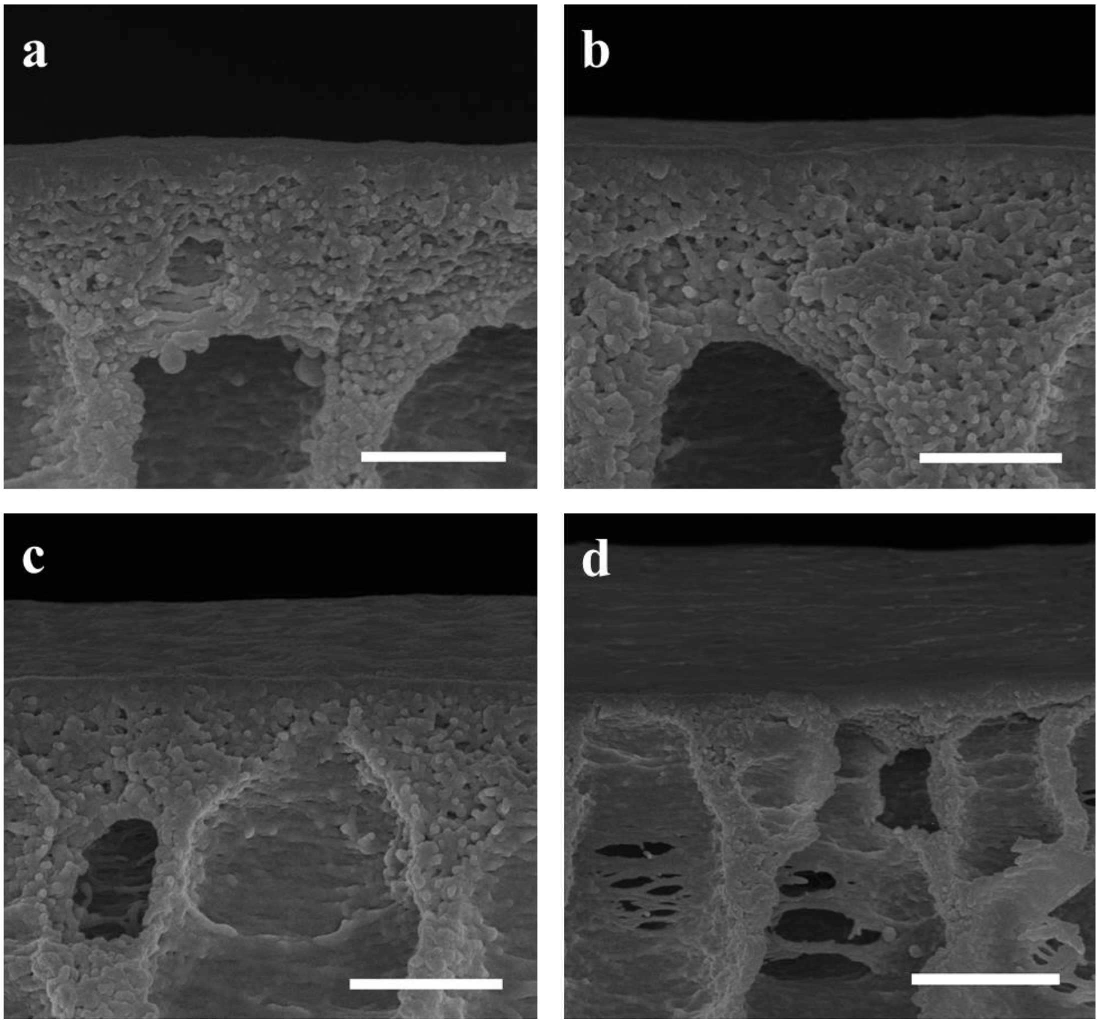

Supplementary Figure 16. Cross-sectional SEM images of the $TpTG_{Cl}$@CNFs-5/PAN membranes with a thickness of a) 65 nm, b) 188 nm, c) 549 nm and d) 2.0 μm (scale bars: **a-c**, 1 μm; **d**, 2 μm).

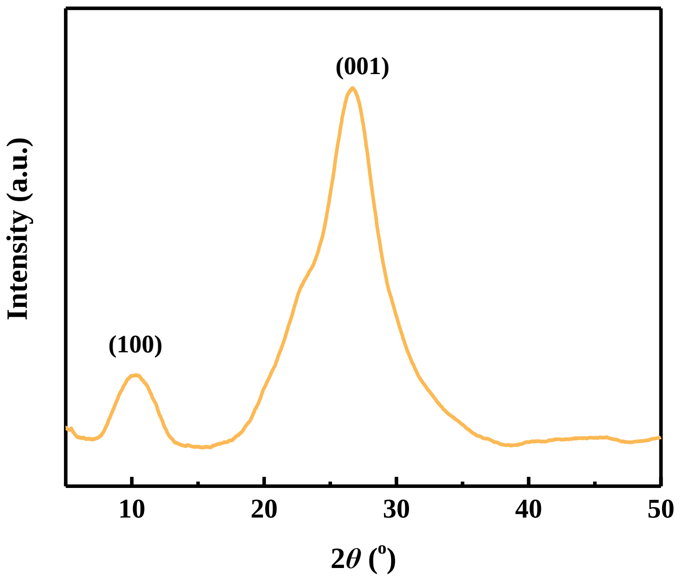

Supplementary Figure 17. PXRD pattern of the freestanding $TpTG_{Cl}$@CNFs-X membrane.

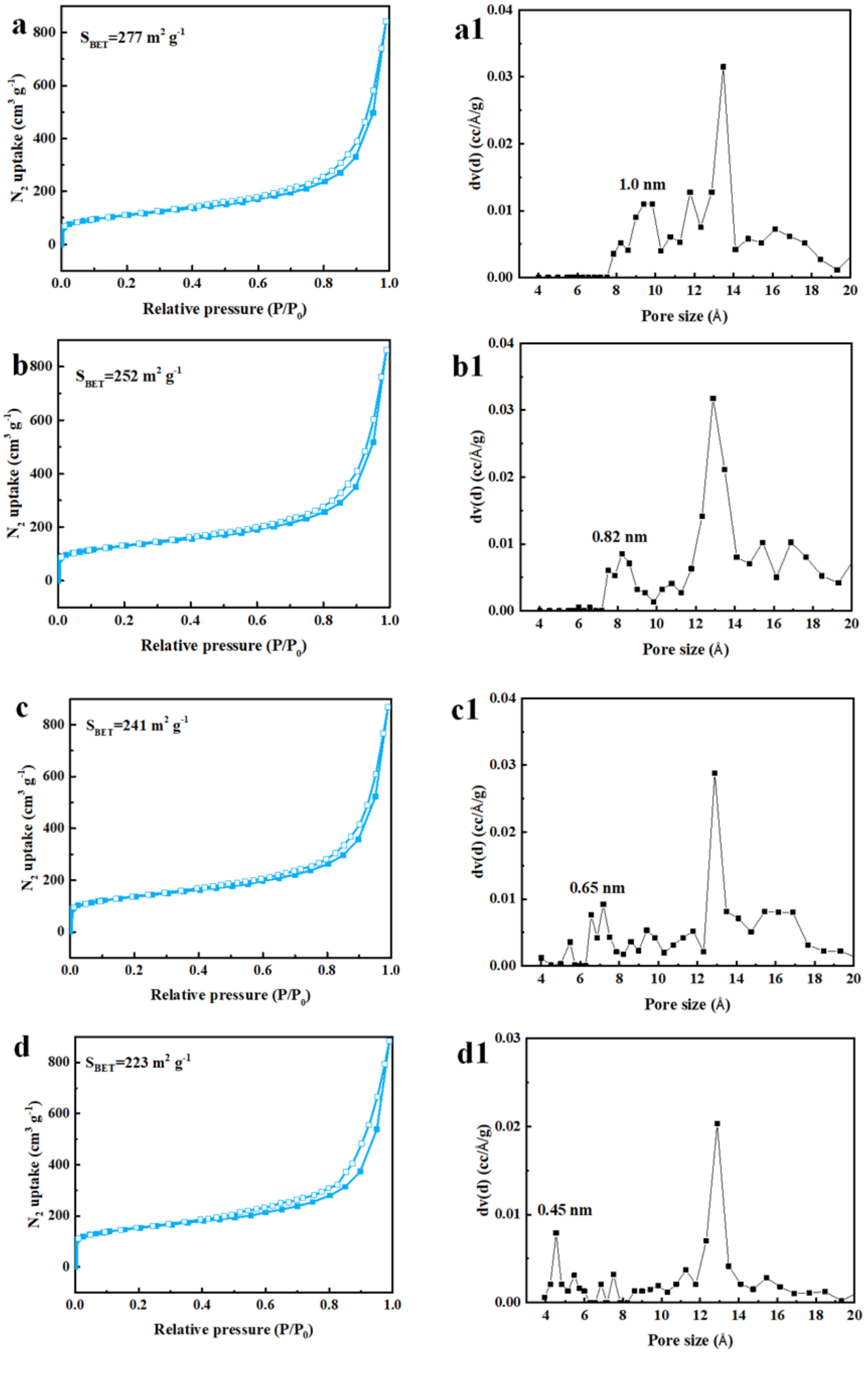

Supplementary Figure 18. Nitrogen adsorption-desorption isotherms measured at 77 K and pore size distribution of the freestanding (a, a1) $TpTG_{Cl}$@CNFs-1, (b, b1) $TpTG_{Cl}$@CNFs-3, (c, c1) $TpTG_{Cl}$@CNFs-4 and (d, d1) $TpTG_{Cl}$@CNFs-5 membranes.

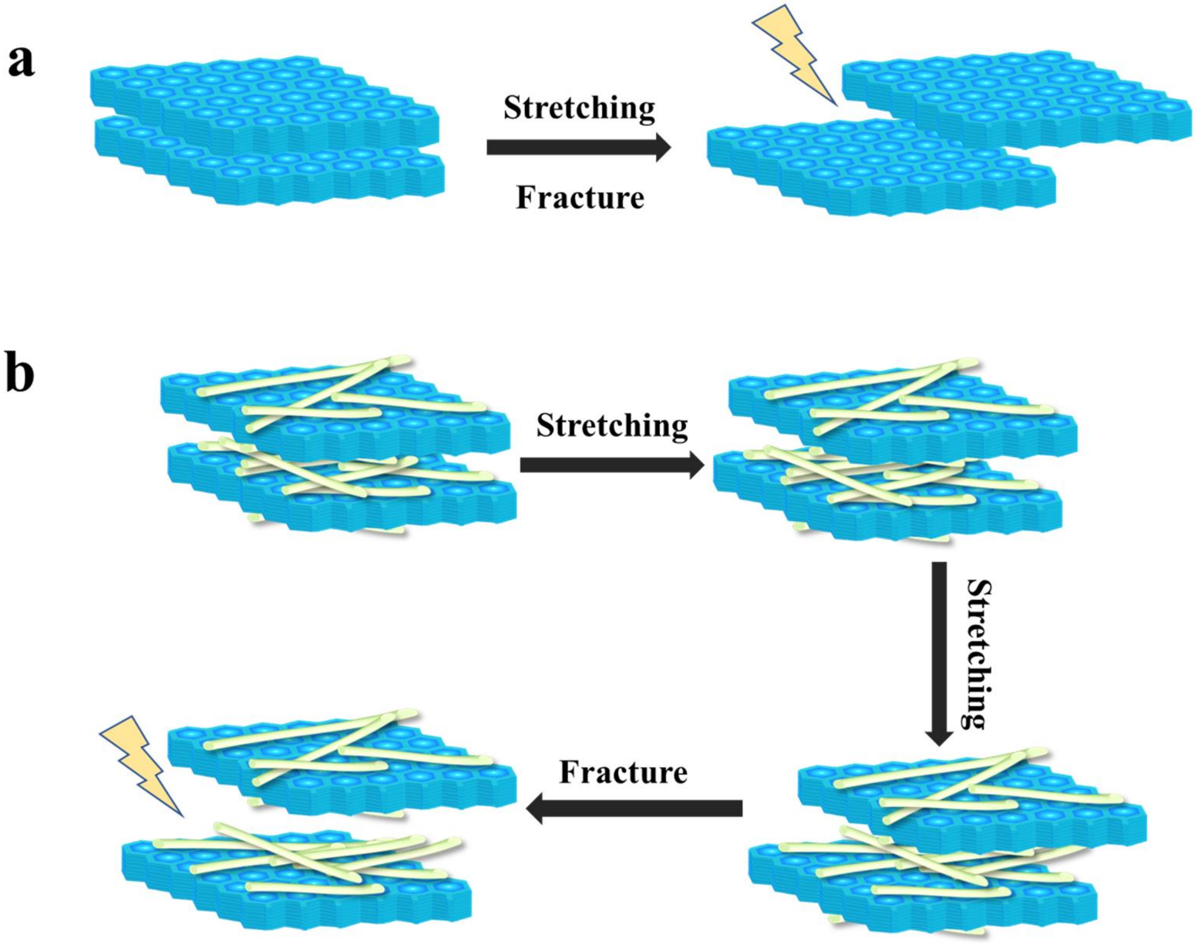

Supplementary Figure 19. Proposed fracture model of the a) pristine $TpTG_{Cl}$ membrane, and b) $TpTG_{Cl}$@CNFs-X membrane.

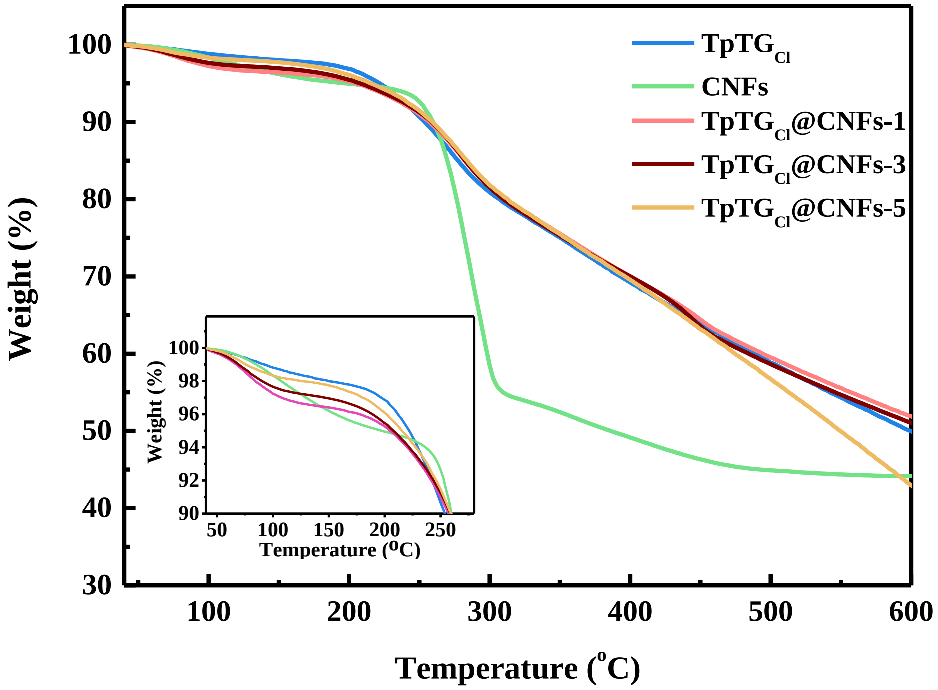

Supplementary Figure 20. TGA curves of the TpTG$_{Cl}$, CNFs and TpTG$_{Cl}$@CNFs-X membranes.

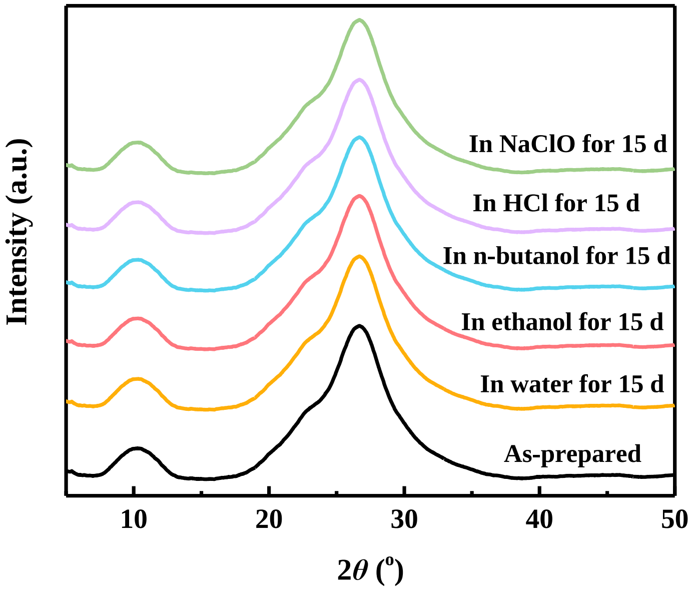

Supplementary Figure 21. PXRD patterns of the $TpTG_{Cl}$@CNFs-5 membranes treated in different solvents for 15 d (the concentration of HCl and NaClO solution is 3 mol L^{-1} and 500 ppm, respectively).

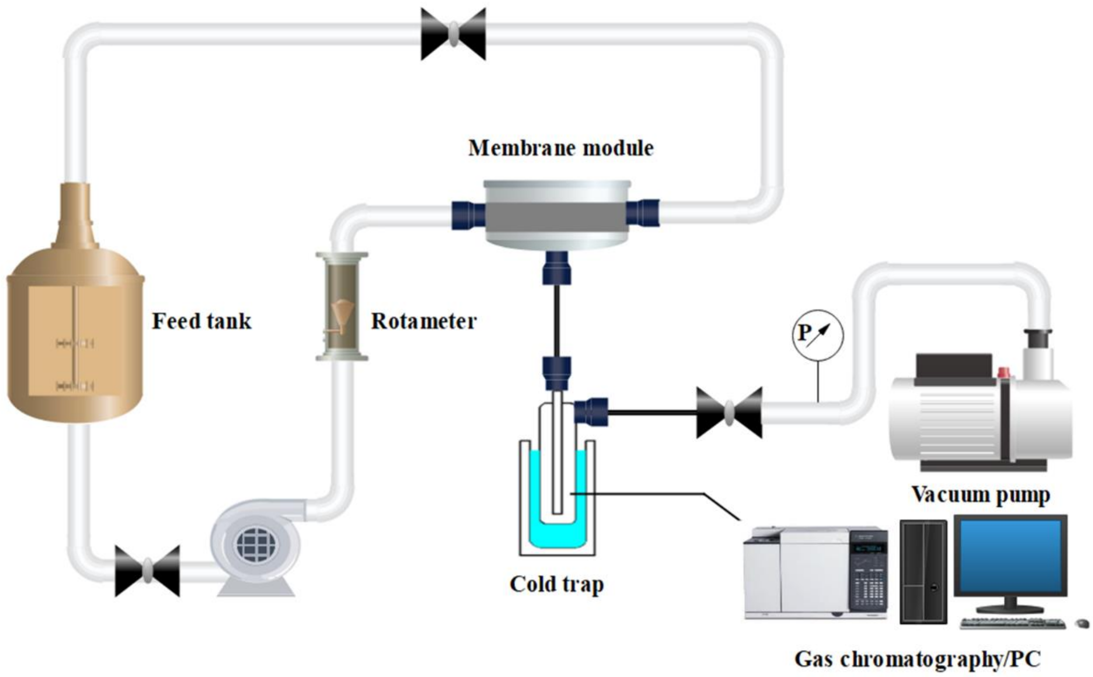

Supplementary Figure 22. Schematic diagram of the pervaporation experiments for water/alcohol separation.

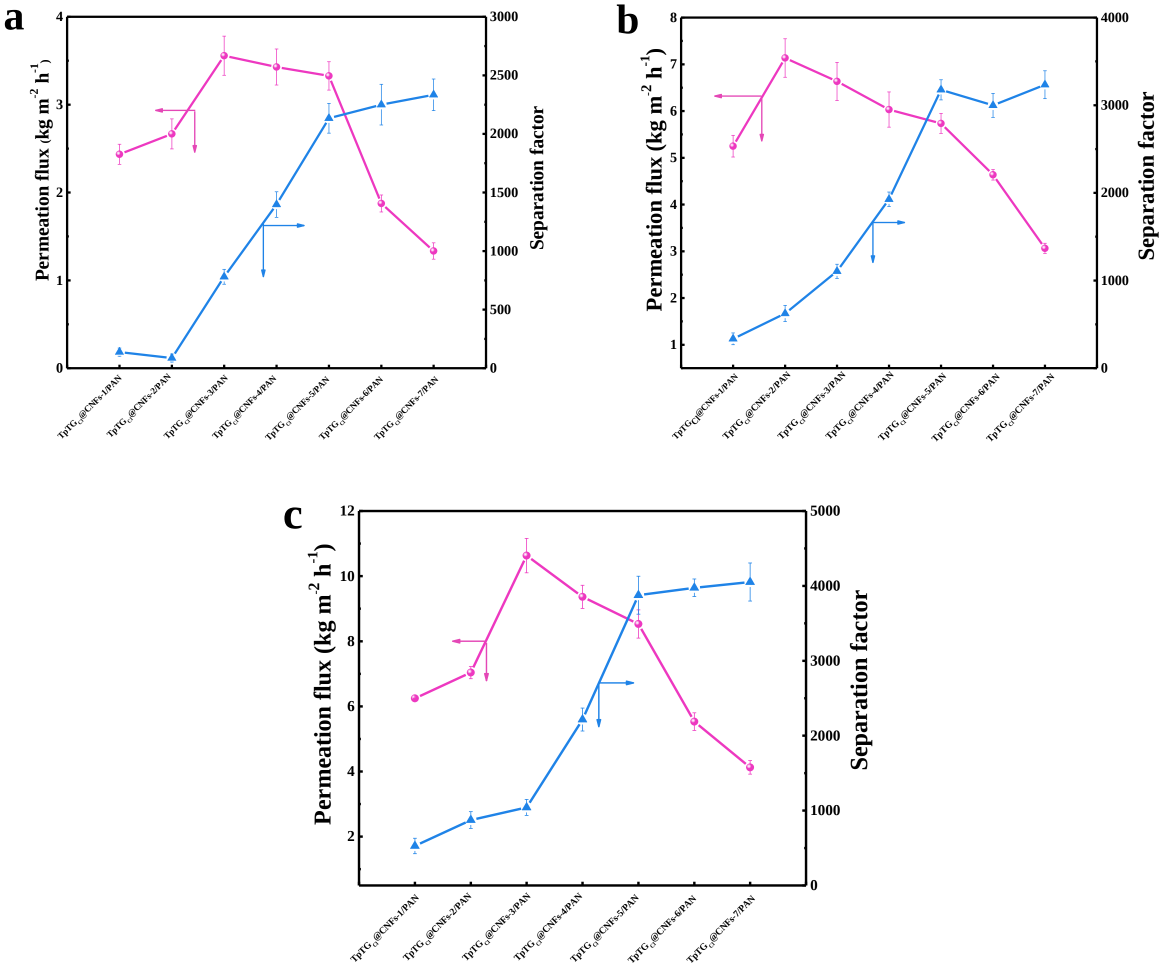

Supplementary Figure 23. a) Ethanol dehydration (at 76 ºC), b) isopropanol dehydration (at 80 ºC), and c) n-butanol dehydration (at 80 ºC) performance of the $TpTG_{Cl}$@CNFs-X/PAN membranes**.** All the membranes were prepared using 30 mL of $TpTG_{Cl}$@CNFs-X solution (0.005 mg mL^{-1}). Error bars represent standard deviations for 3 measurements.

Supplementary Table 2. n-Butanol dehydration performance of the $TpTG_{Cl}$@CNFs-5 membranes as a function of the membrane thickness. Error bars represent standard deviations for 3 measurements.

Thickness	Permeation flux (kg m^{-2} h^{-1})	Separation factor
65 nm	14.37±0.52	235±23
188 nm	8.53±0.43	3876±254
549 nm	6.29±0.39	3920±217
1.2 μm	4.25±0.27	4131±245
2.0 μm	3.14±0.19	4265±262

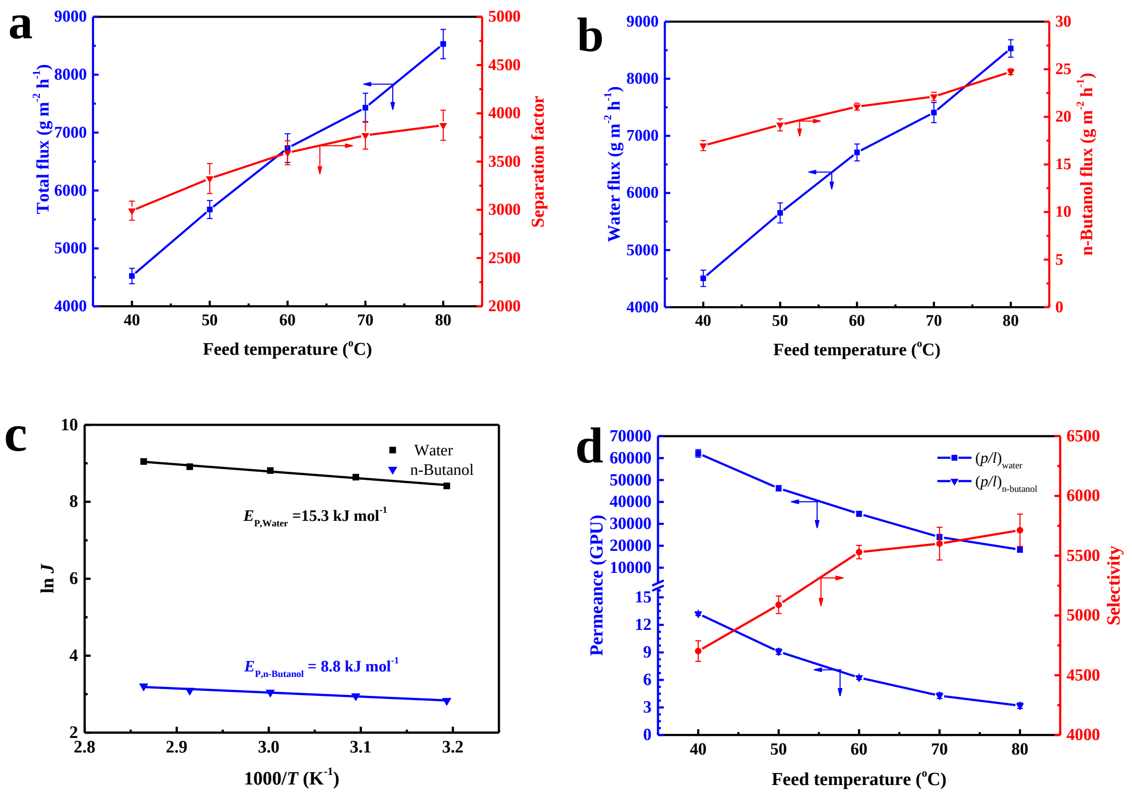

Supplementary Figure 24. Effect of feed temperature on: (a) total flux and separation factor, (b) water and n-butanol flux, (c) Arrhenius plots of water and n-butanol, and (d) permeance and selectivity of the $TpTG_{Cl}$@CNFs-5/PAN membrane for dehydration of 90 wt% n-butanol aqueous solution. Error bars represent standard deviations for 3 measurements.

Note: The activation energy (E_a) of water and n-butanol through the membrane was calculated by the Arrhenius equation. The E_a of water (15.3 kJ mol^{-1}) is much higher than that of n-butanol (8.8 kJ mol^{-1}). Therefore, the increase rate of water flux is higher than that of n-butanol flux with increasing temperature, leading to the increased separation factor at a higher temperature[9, 10]. By eliminating the influence of the driving force of the membrane, the permeance and selectivity of the membrane were calculated as shown in Supplementary Figure 24d. Both the water permeance and n-butanol permeance decline with the increase of feed temperature. Since the dissolution process is exothermic[11], the reduction of dissolution for water and n-butanol plays a

critical role, thus bringing about the decreased permeance with increasing feed temperature. However, the decrease rate for n-butanol permeance is higher than that for water permeance and hence the selectivity is enhanced.

Supplementary Table 3. Comparison of the n-butanol dehydration performance of representative membranes reported in the literatures and in this study.

Membrane type	Temperature (°C)	Water concentration in feed (wt%)	Permeation flux (kg m^{-2} h^{-1})	Separation factor	Reference
GO membrane	70	10	4.34	1791	[12]
MOF membrane	60	15	0.08	3417	[13]
Polymeric membrane	60	10	2.24	1116	[14]
Polymeric membrane	50	5	0.77	481	[15]
Polymeric membrane	60	15	0.39	2518	[16]
Mixed matrix membrane	60	15	0.29	14214	[17]
Polymeric membrane	80	5	0.70	180	[18]
Mixed matrix membrane	80	10	2.54	2735	[19]
Polymeric membrane	60	5	0.25	350	[20]
Polymeric membrane	70	10	1.12	1000	[21]
Polymeric membrane	60	15	0.85	1174	[22]
Polymeric membrane	30	13	2.30	3237	[23]
Silica membrane	60	6	1.50	1000	[24]
Silica membrane	70	5	2.30	680	[25]
Silica membrane	60	5	1.21	2811	[26]
GO membrane	70	10	1.63	5120	[27]
GO membrane	70	10	10.12	1523	[10]
GO membrane	50	10	0.70	15000	[28]
MOF membrane	70	5	5.38	4280	[29]
COF membrane	80	10	8.53	3876	This work

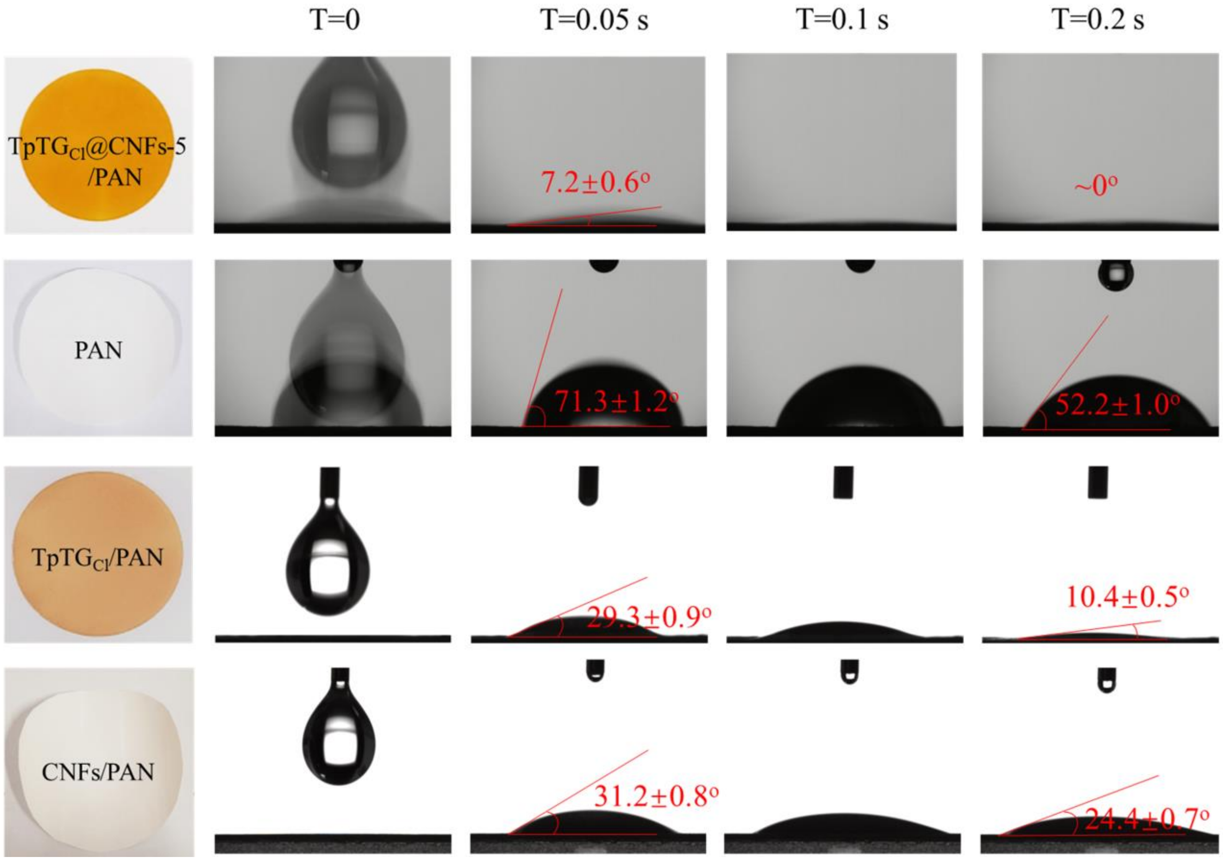

Supplementary Figure 25. Water contact angles of the $TpTG_{Cl}$@CNFs-5/PAN, PAN, pristine $TpTG_{Cl}$/PAN and pristine CNFs/PAN membranes within 0.2 s. Error bars represent standard deviations for 5 measurements.

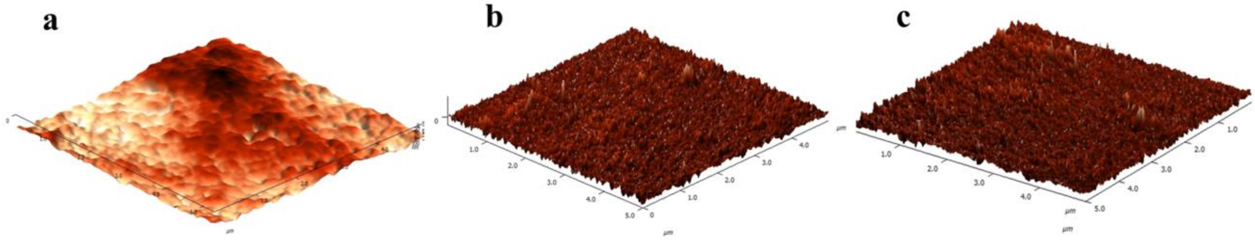

Membrane	Roughness	
	R_a (nm)	R_q (nm)
$TpTG_{Cl}$/PAN	14.8±0.8	18.3±1.1
CNFs/PAN	66.3±1.8	50.6±2.0
$TpTG_{Cl}$@CNFs-5/PAN	69.4±3.7	56.3±2.9

Supplementary Figure 26. 3D AFM images of the a) $TpTG_{Cl}$/PAN, b) CNFs/PAN and c) $TpTG_{Cl}$@CNFs/PAN-5 membrane with a scan area of 5 μm × 5 μm, and the corresponding average roughness (R_a) and root-mean-square roughness (R_q). Error bars represent standard deviations for 3 measurements.

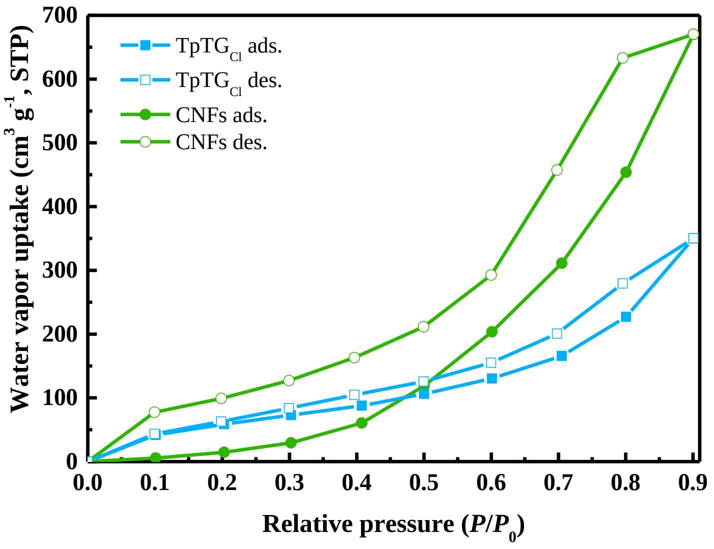

Supplementary Figure 27. Water vapor adsorption-desorption isotherms of the $TpTG_{Cl}$ and CNFs measured at 298 K.

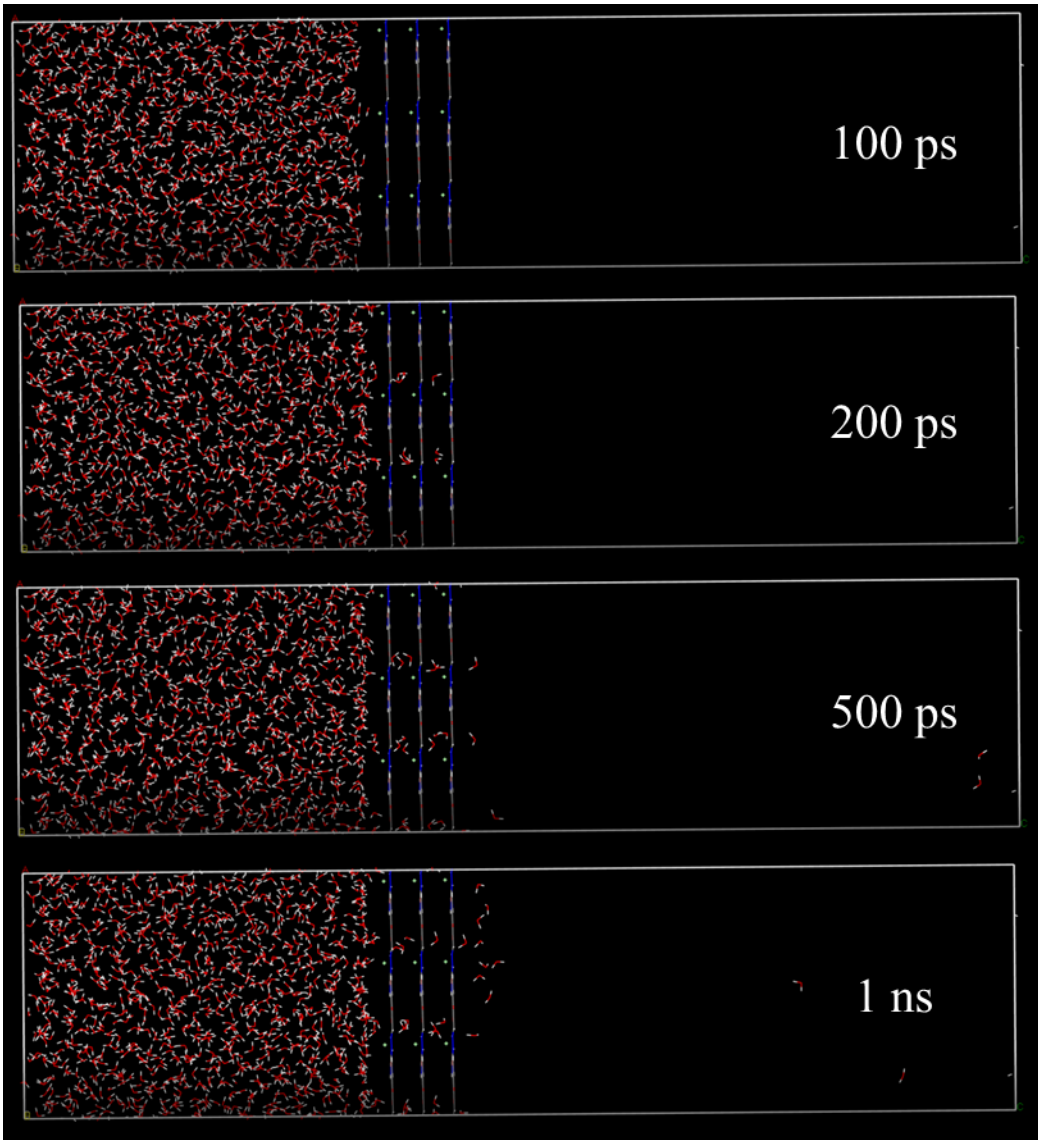

Supplementary Figure 28. Simulation snapshots of water molecules transporting through the $TpTG_{Cl}$ at 100 ps, 200 ps, 500 ps and 1 ns.

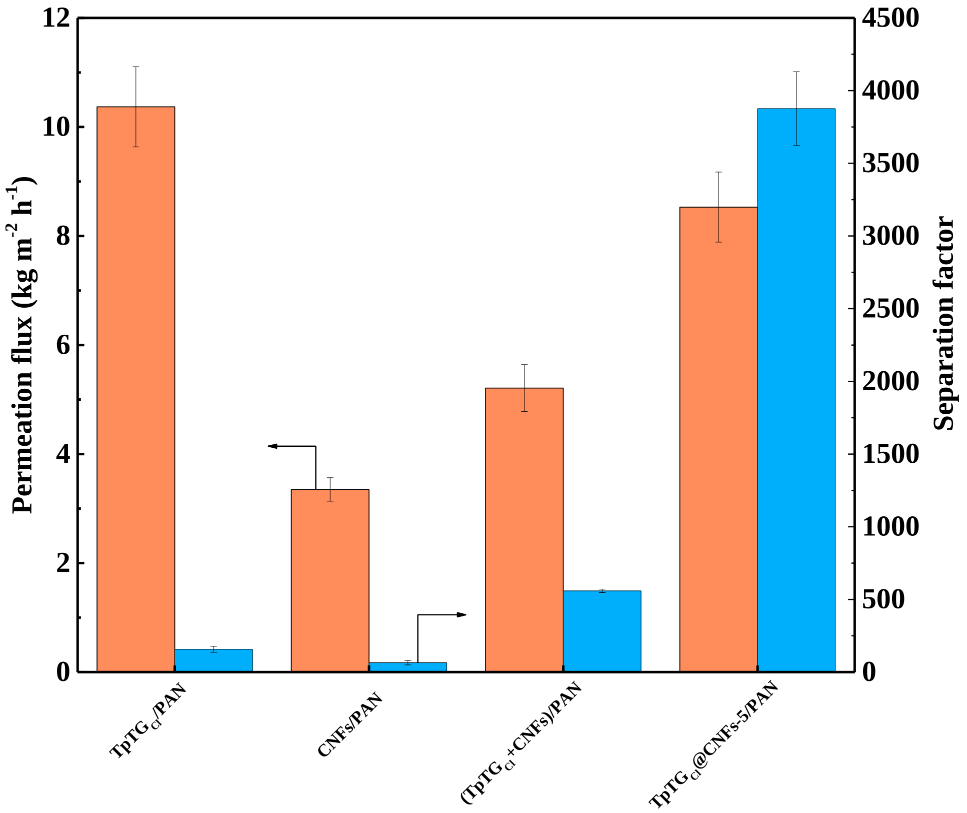

Supplementary Figure 29. Separation performance comparison of the pristine $TpTG_{Cl}$/PAN membrane, pristine CNFs/PAN membrane, ($TpTG_{Cl}$ + CNFs)/PAN membrane and $TpTG_{Cl}$@CNFs-5/PAN membrane for dehydration of 90 wt% n-butanol aqueous solution. Error bars represent standard deviations for 3 measurements.

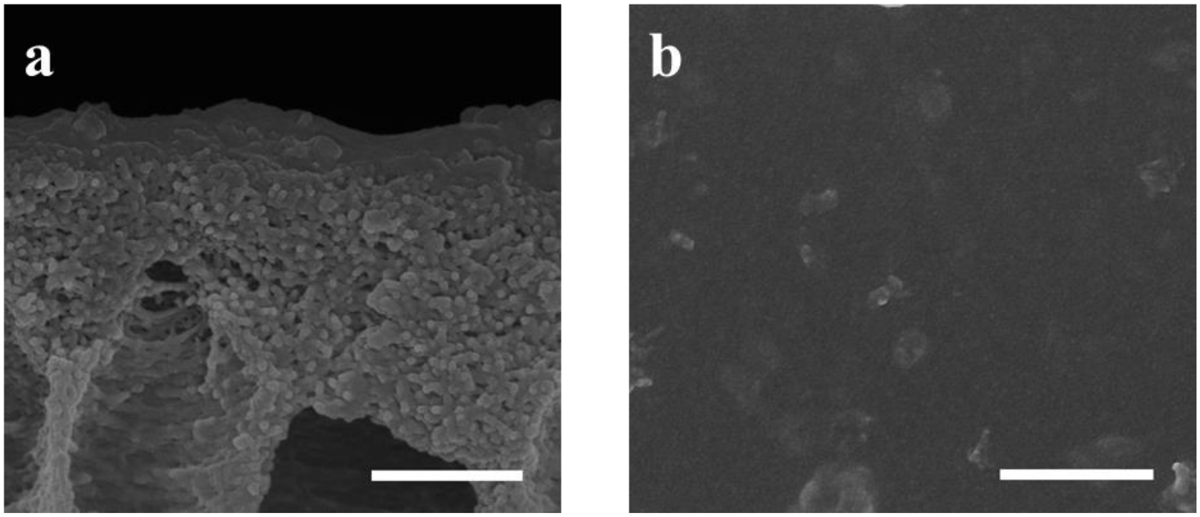

Supplementary Figure 30. a) Cross-sectional and b) surface SEM images of the ($TpTG_{Cl}$ + CNFs)/PAN membrane (scale bar: **a** and **b**, 1 μm).

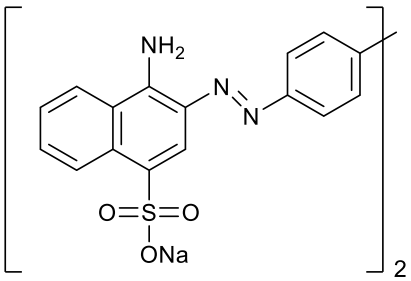

Molecular weight: 696
Maximum absorption wavelength: 490 nm
Diameter: 2.20 nm
Charge: -2
Congo red

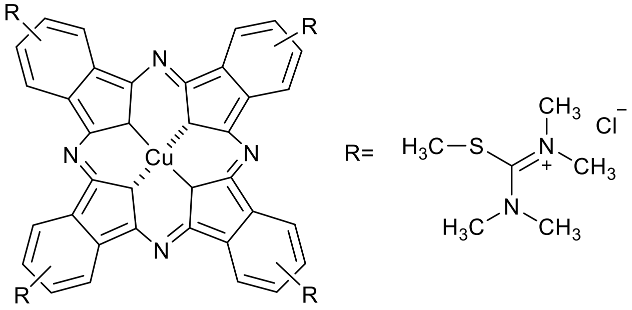

Molecular weight: 1299
Maximum absorption wavelength: 331 nm
Diameter: 2.22 nm
Charge: +1
Alcian blue

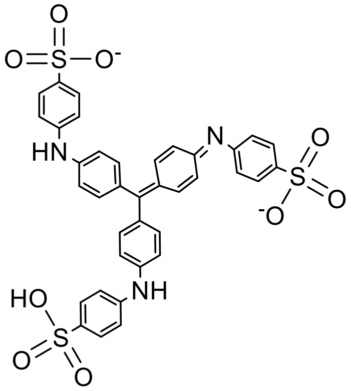

Molecular weight: 799
Maximum absorption wavelength: 600 nm
Diameter: 2.06 nm
Charge: -2
Methyl blue

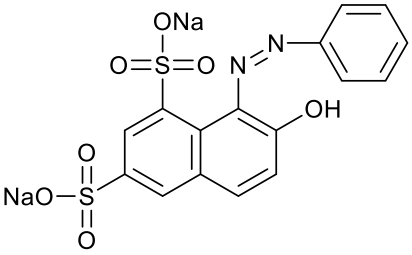

Molecular weight: 452
Maximum absorption wavelength: 485 nm
Diameter: 1.08 nm
Charge: -1
Orange GII

Supplementary Figure 31. The characteristics of the organic dyes used in this work.

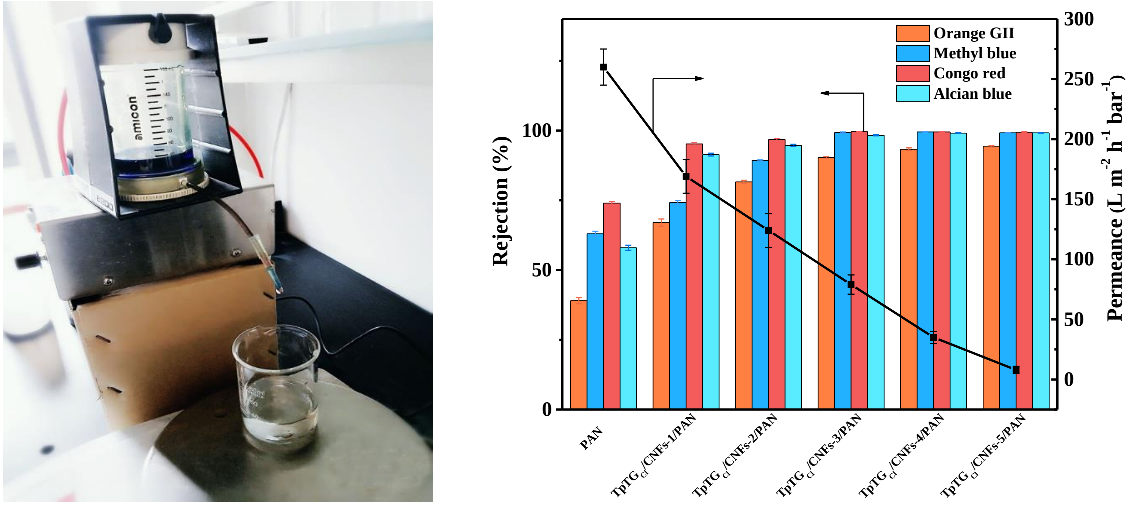

Supplementary Figure 32. Nanofiltration experiments and the dye rejection performance of the membranes. All the membranes were prepared using 30 mL of $TpTG_{Cl}$@CNFs-X solution (0.005 mg mL^{-1}). Error bars represent standard deviations for 3 measurements.

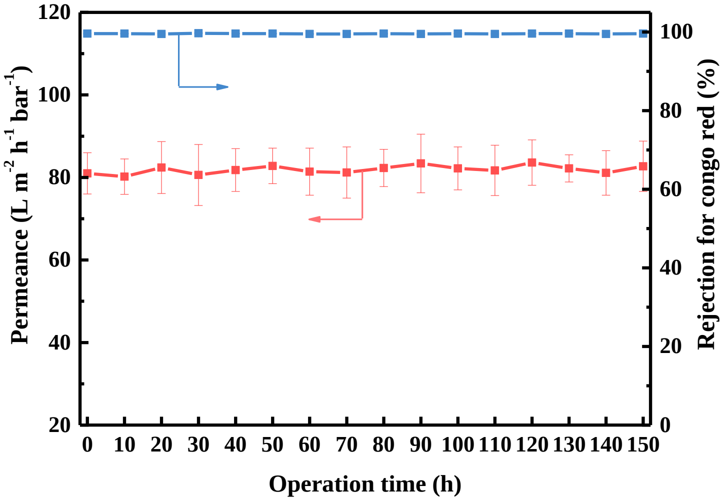

Supplementary Figure 33. Long-term stability of the $TpTG_{Cl}$@CNFs-3/PAN membrane for rejecting congo red (dye concentration: 100 ppm; applied pressure: 0.2 MPa; room temperature). Error bars represent standard deviations for 3 measurements.

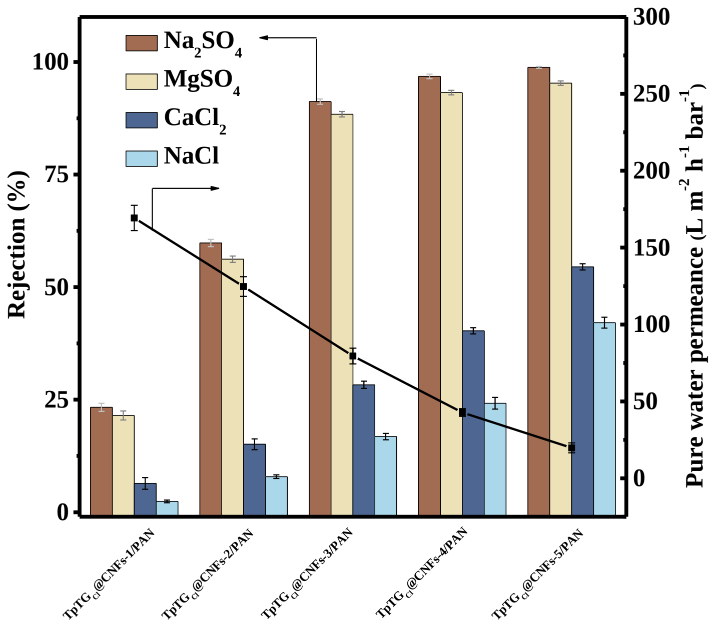

Supplementary Figure 34. The salt rejection performance of the $TpTG_{Cl}$@CNFs-X/PAN membranes. All the membranes were prepared using 30 mL of $TpTG_{Cl}$@CNFs-X solution (0.005 mg mL^{-1}). Error bars represent standard deviations for 3 measurements.

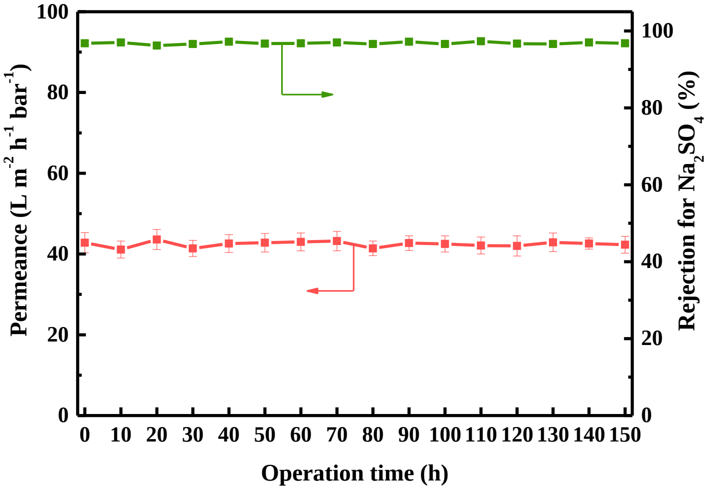

Supplementary Figure 35. Long-term stability of the $TpTG_{Cl}$@CNFs-4/PAN membrane for rejecting Na_2SO_4 (salt concentration: 1000 ppm; applied pressure: 0.4 MPa; room temperature). Error bars represent standard deviations for 3 measurements.

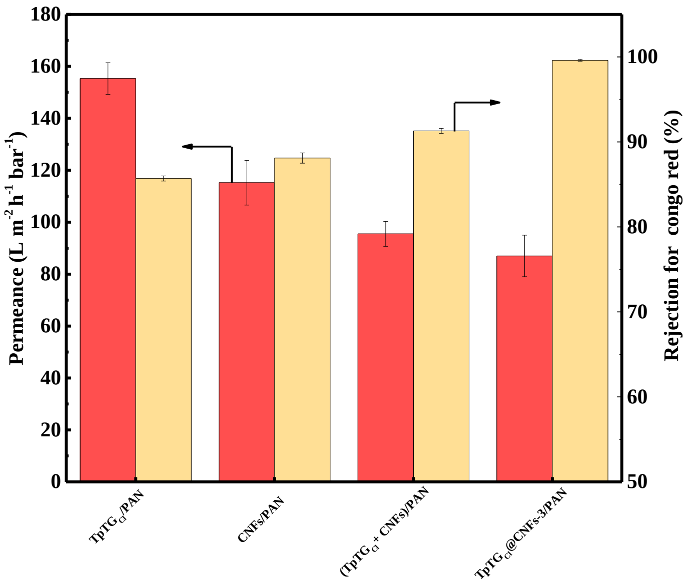

Supplementary Figure 36. Nanofiltration performance comparison of the pristine $TpTG_{Cl}$/PAN membrane, pristine CNFs/PAN membrane, ($TpTG_{Cl}$ + CNFs)/PAN membrane and $TpTG_{Cl}$@CNFs-3/PAN membrane for rejecting congo red. Error bars represent standard deviations for 3 measurements.

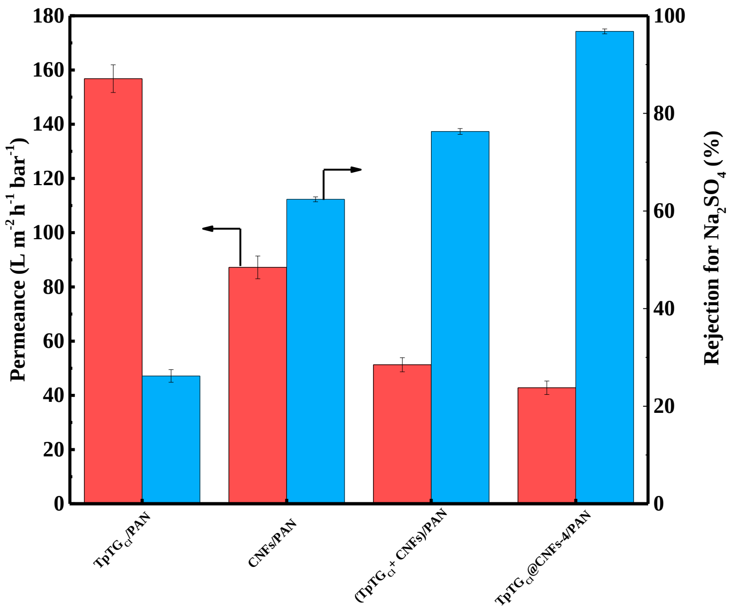

Supplementary Figure 37. Nanofiltration performance comparison of the pristine $TpTG_{Cl}$/PAN membrane, pristine CNFs/PAN membrane, ($TpTG_{Cl}$ + CNFs)/PAN membrane and $TpTG_{Cl}$@CNFs-4/PAN membrane for rejecting Na_2SO_4. Error bars represent standard deviations for 3 measurements.

Supplementary Table 4. Comparison of the Na_2SO_4 rejection performance of representative nanofiltration membranes reported in the literatures and in this study.

Membrane type	Applied pressure (bar)	Na_2SO_4 concentration in feed (wt%)	Permeance (L m^{-2} h^{-1} bar^{-1})	Rejection (%)	Reference
Thin film composite membrane	6	0.2	7.8	97.4	[30]
Thin film composite membrane	6	0.071	5.4	80	[31]
Thin film composite membrane	6	0.1	13.2	96.8	[32]
Thin film composite membrane	6	0.2	6.4	92	[33]
GO membrane	15	0.2	2.7	95	[34]
GO membrane	5	0.284	21	60	[35]
GO membrane	8	0.2	9.3	87.7	[36]
GO membrane	5	0.142	11.3	83.5	[37]
Polyamide membrane	6	0.2	4.0	94.1	[38]
Polyamide membrane	6	0.2	7.4	98.1	[39]
Polyamide membrane	6	0.2	25.6	92.1	[40]
Polyamide membrane	5	0.05	16.4	97.5	[41]
Polyamide membrane	6	0.2	13.2	99.5	[42]
Polyamide membrane	6	0.1	32	95.9	[43]
Polyamide membrane	6	0.1	17.6	95	[44]
Polyamide membrane	6	0.1	7.5	96.4	[45]
Polyamide membrane	4	0.05	18	70	[46]
Polyamide membrane	6	0.1	34	97	[47]
Polyamide membrane	4	0.1	53.5	95.2	[48]
Polyamide membrane	4	0.15	25.1	99.1	[49]
Polyamide membrane	4.8	0.2	24.8	99.6	[50]
COF membrane	4	0.1	42.8	96.8	This work

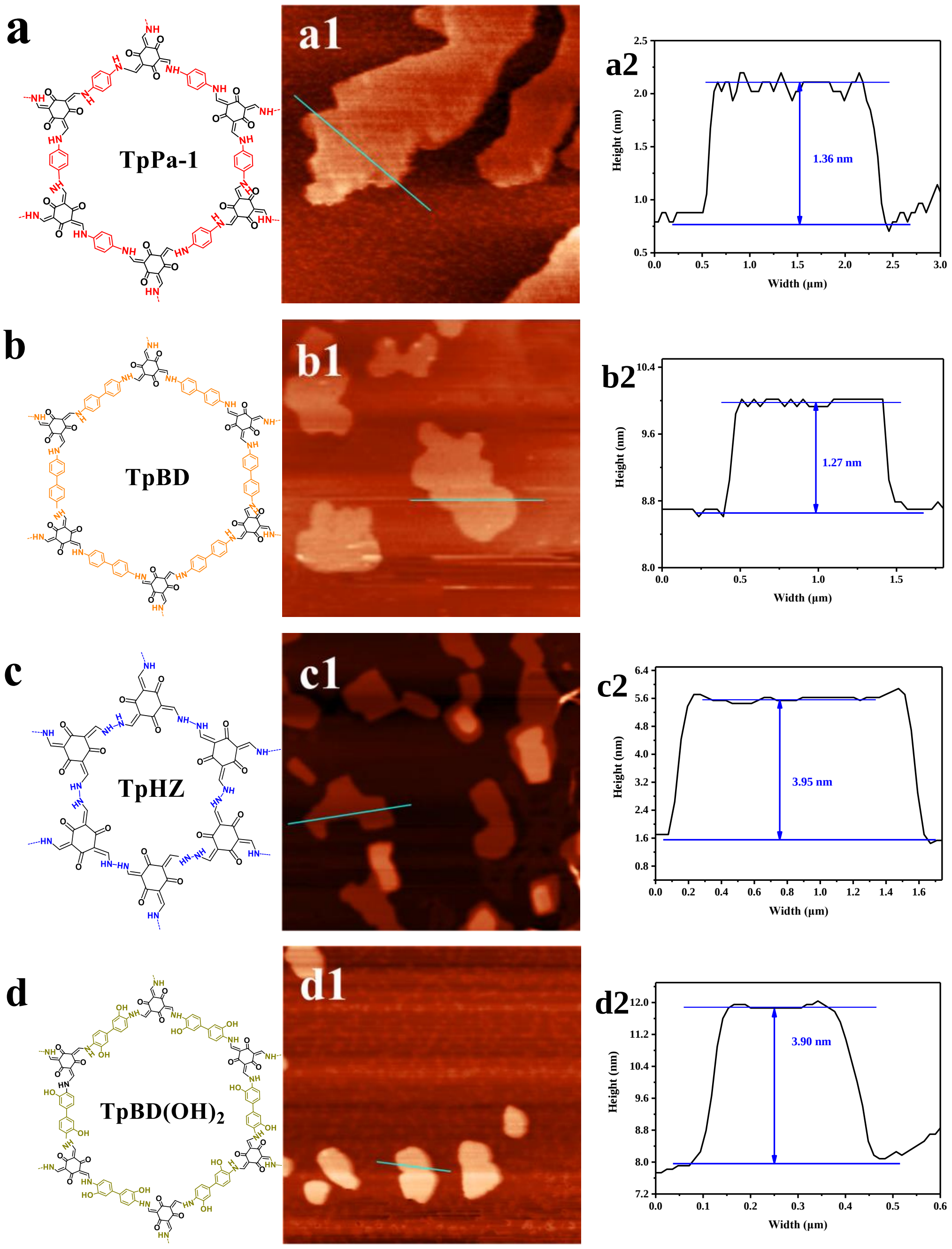

Supplementary Figure 38. Molecular structures, AFM images and height profiles of (a, a1, a2) COF TpPa-1, (b, b1, b2) TpBD, (c, c1, c2) TpHZ and (d, d1, d2) $TpBD(OH)_2$.

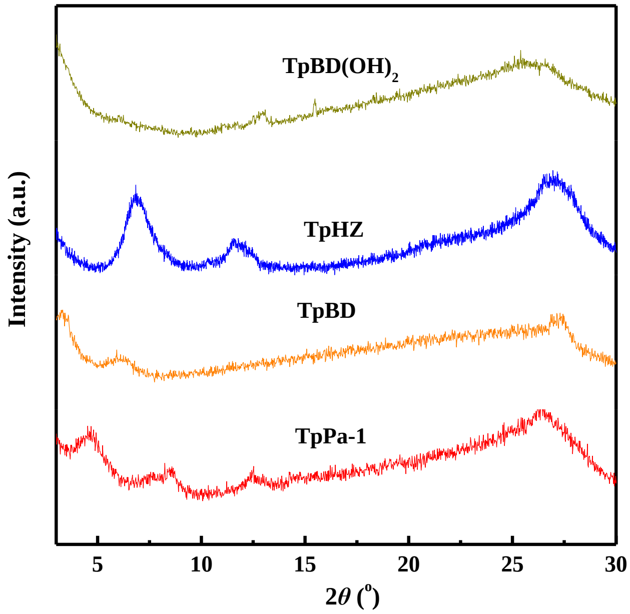

Supplementary Figure 39. PXRD patterns of the exfoliated COF TpPa-1, TpBD, TpHZ and $TpBD(OH)_2$.

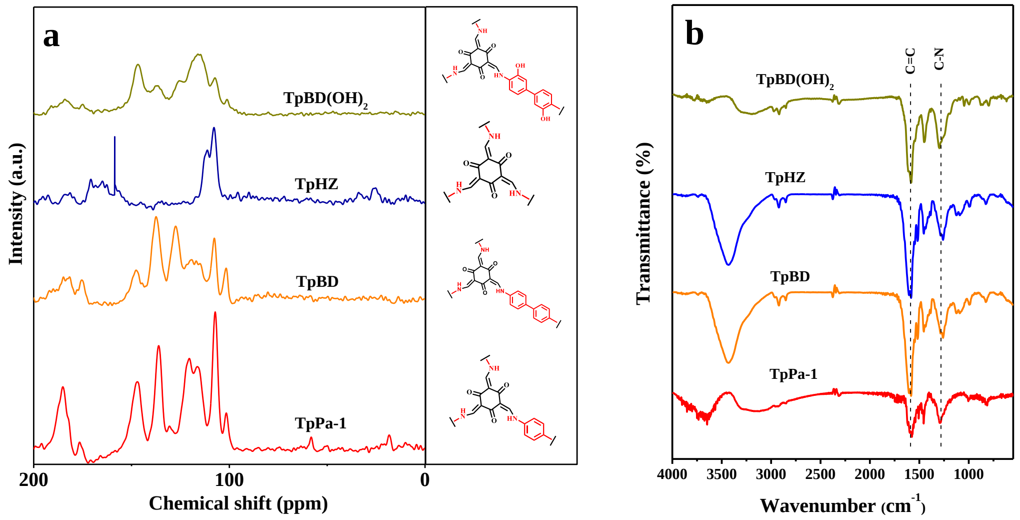

Supplementary Figure 40. a) ^{13}C CP-MAS solid-state NMR spectra and b) FTIR spectra of COF TpHZ, TpBD, TpPa-1 and $TpBD(OH)_2$.

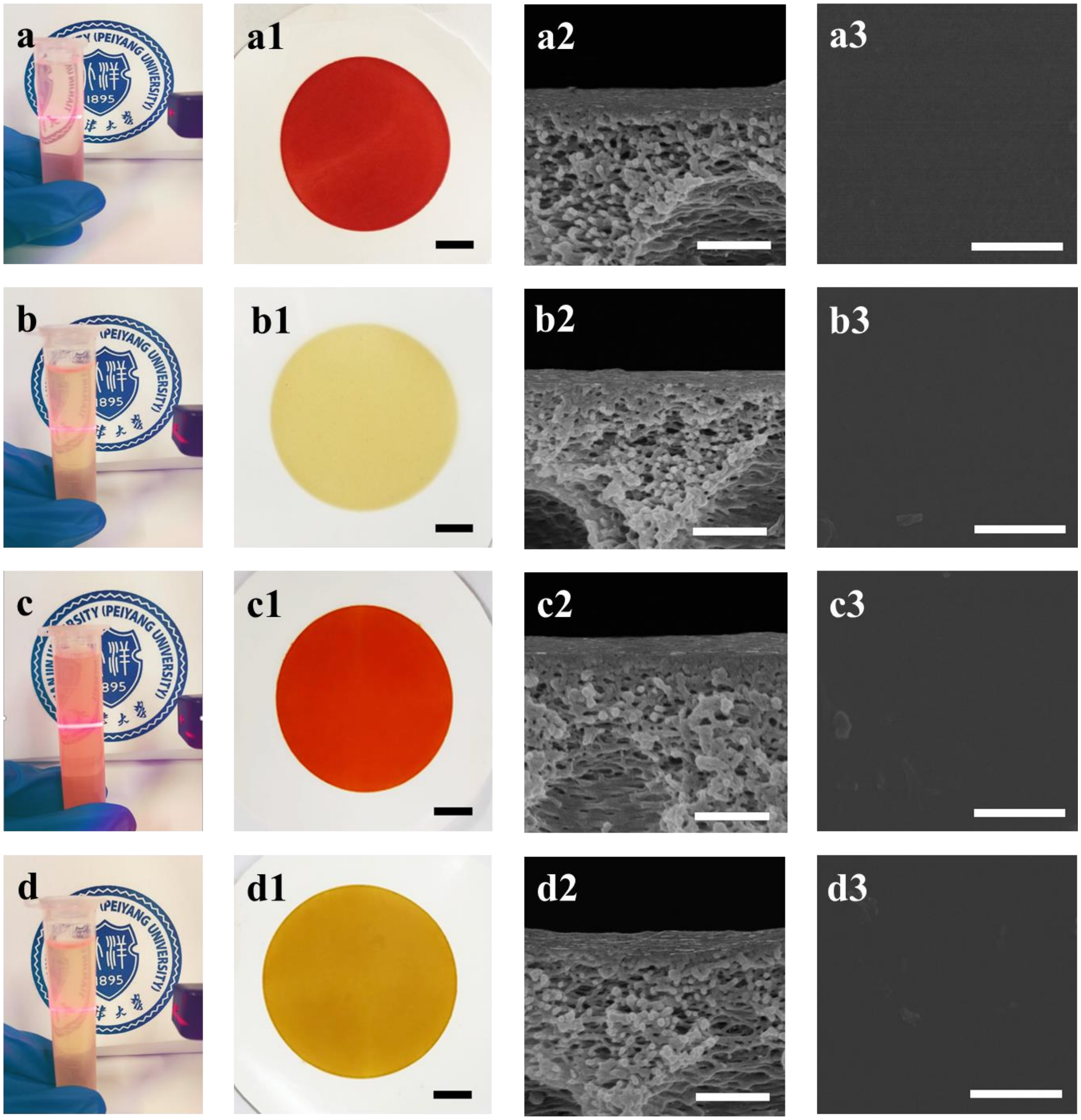

Supplementary Figure 41. Tyndall scattering effect in the aqueous solution of (a) TpPa-1@CNFs, (b) TpBD@CNFs, (c) TpHZ@CNFs and (d) TpBD$(OH)_2$@CNFs nanocomposites; The digital photos, cross-sectional and surface SEM images of (a1, a2, a3) TpPa-1@CNFs/PAN membrane, (b1, b2, b3) TpBD@CNFs/PAN membrane, (c1, c2, c3) TpHZ@CNFs/PAN membrane and (d1, d2, d3) TpBD$(OH)_2$@CNFs/PAN membrane (scale bar: **a1-d1**, 1 cm; **a2-d2**, 1 μm; **a3-d3**, 10 μm)

Supplementary Table 5. Mechanical properties of the four kinds of COFs@CNFs-X membranes and the corresponding pristine COF membranes. Error bars represent standard deviations for 3 measurements.

Membrane	Tensile strength (MPa)	Young's modulus (GPa)
TpPa-1@CNFs-5	112.3±5.1	4.2±0.1
TpBD@CNFs-5	95.6±4.4	3.8±0.2
TpHZ@CNFs-5	107.5±6.8	4.2±0.1
$TpBD(OH)_2$@CNFs-5	98.2±6.1	4.0±0.2
TpPa-1	20.8±0.7	1.8±0.1
TpBD	21.2±0.5	2.0±0.1
TpHZ	14.6±0.3	1.7±0.1
$TpBD(OH)_2$	25.3±0.4	2.0±0.2

Supplementary Table 6. Nanofiltration performance of the four kinds of COFs@CNFs-X/PAN membranes. Error bars represent standard deviations for 3 measurements.

Membrane	Permeance ($L\ m^{-2}\ h^{-1}\ bar^{-1}$)	Rejection for Na_2SO_4 (%)	Rejection for congo red (%)
TpHZ@CNFs-5/PAN	47±6	91.4±1.2	99.8±0.1
TpBD@CNFs-5/PAN	315±32	0.8±0.1	71.4±0.3
TpPa-1@CNFs-5/PAN	117±18	5.5±0.3	99.3±0.1
$TpBD(OH)_2$@CNFs-5/PAN	225±33	3.2±0.2	85.2±0.4

Note: All the membranes were fabricated by filtrating 30 mL of COFs@CNFs-5 solution (0.005 mg mL^{-1}) on PAN substrates.

References

1. Chong, J. H., Sauer, M., Patrick, B. O. & MacLachlan, M. J. Highly stable keto-enamine salicylideneanilines. *Org. Lett.* **5**, 3823-3826 (2003).
2. Mitra, S. et al. Self-exfoliated guanidinium-based ionic covalent organic nanosheets (iCONs). *J. Am. Chem. Soc.* **138**, 2823-2828 (2016).
3. Isogai, A., Saito, T. & Fukuzumi, H. TEMPO-oxidized cellulose nanofibers. *Nanoscale* **3**, 71-85 (2011).
4. Kang, Z. et al. Mixed matrix membranes (MMMs) comprising exfoliated 2D covalent organic frameworks (COFs) for efficient CO_2 separation. *Chem. Mater.* **28**, 1277-1285 (2016).
5. Chandra, S. et al. Chemically stable multilayered covalent organic nanosheets from covalent organic frameworks via mechanical delamination. *J. Am. Chem. Soc.* **135**, 17853-17861 (2013).
6. Karak, S., Kumar, S., Pachfule, P. & Banerjee, R. Porosity prediction through hydrogen bonding in covalent organic frameworks. *J. Am. Chem. Soc.* **140**, 5138-5145 (2018).
7. Yang, X. M., Tu, Y. F., Li, L. A., Shang, S. M. & Tao, X. M. Well-dispersed chitosan/graphene oxide nanocomposites. *ACS Appl. Mater. Interfaces* **2**, 1707-1713 (2010).
8. Shen, J. et al. Subnanometer two-dimensional graphene oxide channels for ultrafast gas sieving. *ACS Nano* **10**, 3398-3409 (2016).
9. Cao, K. et al. Enhanced water permeation through sodium alginate membranes by incorporating graphene oxides. *J. Membr. Sci.* **469**, 272-283 (2014).

10. Huang, K. et al. High-efficiency water-transport channels using the synergistic effect of a hydrophilic polymer and graphene oxide laminates. *Adv. Funct. Mater.* **25**, 5809-5815 (2015).

11. Cheng, X. X. et al. Water-selective permeation in hybrid membrane incorporating multi-functional hollow ZIF-8 nanospheres. *J. Membr. Sci.* **555**, 146-156 (2018).

12. Tsou, C. H. et al. Effect of microstructure of graphene oxide fabricated through different self-assembly techniques on 1-butanol dehydration. *J. Membr. Sci.* **477**, 93-100 (2015).

13. Shi, G. M., Yang, T. X. & Chung, T. S. Polybenzimidazole (PBI)/zeolitic imidazolate frameworks (ZIF-8) mixed matrix membranes for pervaporation dehydration of alcohols. *J. Membr. Sci.* **415**, 577-586 (2012).

14. Liu, T. et al. Preparation and characterization of polyelectrolyte complex membranes bearing alkyl side chains for the pervaporation dehydration of alcohols. *J. Membr. Sci.* **429**, 181-189 (2013).

15. Zhang, G. J., Song, X., Ji, S. L., Wang, N. X. & Liu, Z. Z. Self-assembly of inner skin hollow fiber polyelectrolyte multilayer membranes by a dynamic negative pressure layer-by-layer technique. *J. Membr. Sci.* **325**, 109-116 (2008).

16. Widjojo, N. & Chung, T. S. Pervaporation dehydration of C2-C4 alcohols by 6FDA-ODA-NDA/Ultem dual-layer hollow fiber membranes with enhanced separation performance and swelling resistance. *Chem. Eng. J.* **155**, 736-743 (2009).

17. Xu, Y. M. & Chung, T. S. High-performance UiO-66/polyimide mixed matrix membranes for ethanol, isopropanol and n-butanol dehydration via pervaporation. *J. Membr. Sci.* **531**, 16-26 (2017).

18. Guo, W. F., Chung, T. S. & Matsuuraa, T. Pervaporation study on the dehydration of aqueous butanol solutions: a comparison of flux vs. permeance, separation factor vs. selectivity. *J. Membr. Sci.* **245**, 199-210 (2004).

19. Yang, H. et al. Hierarchical pore architectures from 2D covalent organic nanosheets for efficient water/alcohol separation. *J. Membr. Sci.* **561**, 79-88 (2018).

20. Schehlmann M. S., W. E. Pervaporation and vapor permeation at the azeotropic point or in the vicinity of the LLE boundary phases of organic/aqueous mixtures. *J. Membr. Sci.* **107**, 277-282 (1995).

21. Zhu, Y. X., Xia, S. S., Liu, G. P. & Jin, W. Q. Preparation of ceramic-supported poly(vinyl alcohol)-chitosan composite membranes and their applications in pervaporation dehydration of organic/water mixtures. *J. Membr. Sci.* **349**, 341-348 (2010).

22. Wang, Y., Goh, S. H., Chung, T. S. & Na, P. Polyamide-imide/polyetherimide dual-layer hollow fiber membranes for pervaporation dehydration of C1-C4 alcohols. *J. Membr. Sci.* **326**, 222-233 (2009).

23. Kalyani, S., Smitha, B., Sridhar, S. & Krishnaiah, A. Blend membranes of sodium alginate and hydroxyethylcellulose for pervaporation-based enrichment of t-butyl alcohol. *Carbohydr. Polym.* **64**, 425-432 (2006).

24. Ben, B. et al. Pervaporation of binary water-alcohol and methanol-alcohol mixtures through microporous methylated silica membranes: Maxwell-Stefan modeling. *Comput. Chem. Eng.* **34**, 1775-1788 (2010).

25. Verkerk, A. W., van Male, P., Vorstman, M. A. G. & Keurentjes, J. T. F. Properties of high flux ceramic pervaporation membranes for dehydration of alcohol/water mixtures. *Sep. Purif. Technol.* **22-3**, 689-695 (2001).

26. Huang, B. X., Liu, Q., Caro, J. & Huang, A. S. Iso-butanol dehydration by pervaporation using zeolite LTA membranes prepared on 3-aminopropyltriethoxysilane-modified alumina tubes. *J. Membr. Sci.* **455**, 200-206 (2014).

27. Li, G. H., Shi, L., Zeng, G. F., Zhang, Y. F. & Sun, Y. H. Efficient dehydration of the organic solvents through graphene oxide (GO)/ceramic composite membranes. *RSC Adv.* **4**, 52012-52015 (2014).

28. Yang, J. J. et al. Self-assembly of thiourea-crosslinked graphene oxide framework membranes toward separation of small molecules. *Adv. Mater.* **30**, 1705775 (2018).

29. Liu, X. L., Wang, C. H., Wang, B. & Li, K. Novel organic-dehydration membranes prepared from zirconium metal-organic frameworks. *Adv. Funct. Mater.* **27**, 1604311 (2017).

30. Wu, H. Q., Tang, B. B. & Wu, P. Y. Optimizing polyamide thin film composite membrane covalently bonded with modified mesoporous silica nanoparticles. *J. Membr. Sci.* **428**, 341-348 (2013).

31. Hu, D., Xu, Z. L. & Chen, C. Polypiperazine-amide nanofiltration membrane containing silica nanoparticles prepared by interfacial polymerization. *Desalination* **301**, 75-81 (2012).

32. Zheng, J. F. et al. Sulfonated multiwall carbon nanotubes assisted thin-film nanocomposite membrane with enhanced water flux and anti-fouling property. *J. Membr. Sci.* **524**, 344-353 (2017).

33. Tang, Y. J., Xu, Z. L., Huang, B. Q., Wei, Y. M. & Yang, H. Novel polyamide thin-film composite nanofiltration membrane modified with poly(amidoamine) and SiO_2 gel. *RSC Adv.* **6**, 45585-45594 (2016).

34. Bano, S., Mahmood, A., Kim, S. J. & Lee, K. H. Graphene oxide modified polyamide nanofiltration membrane with improved flux and antifouling properties. *J. Mater. Chem. A* **3**, 2065-2071 (2015).

35. Han, Y., Xu, Z. & Gao, C. Ultrathin graphene nanofiltration membrane for water purification. *Adv. Funct. Mater.* **23**, 3693-3700 (2013).

36. Xu, X. L. et al. Graphene oxide nanofiltration membranes stabilized by cationic porphyrin for high salt rejection. *ACS Appl. Mater. Interfaces* **8**, 12588-12593 (2016).

37. Han, Y., Jiang, Y. Q. & Gao, C. High-flux graphene oxide nanofiltration membrane intercalated by carbon nanotubes. *ACS Appl. Mater. Interfaces* **7**, 8147-8155 (2015).

38. Chen, G. E. et al. Preparation and characterization of a composite nanofiltration membrane from cyclen and trimesoyl chloride prepared by interfacial polymerization. *J. Appl. Polym. Sci.* **132**, 42345 (2015).

39. Chen, G. E. et al. Fabrication and characterization of a novel nanofiltration membrane by the interfacial polymerization of 1,4-diaminocyclohexane (DCH) and trimesoyl chloride (TMC). *RSC Adv.* **5**, 40742-40752 (2015).

40. Tang, Y. J., Wang, L. J., Xu, Z. L., Wei, Y. M. & Yang, H. Novel high-flux thin film composite nanofiltration membranes fabricated by the NaClO pre-oxidation of the mixed diamine monomers of PIP and BHTTM in the aqueous phase solution. *J. Membr. Sci.* **502**, 106-115 (2016).

41. Pan, Y. Y. et al. Enhanced both perm-selectivity and fouling resistance of poly(piperazine-amide) nanofiltration membrane by incorporating sericin as a co-reactant of aqueous phase. *J. Membr. Sci.* **523**, 282-290 (2017).

42. Tang, Y. J., Xu, Z. L., Xue, S. M., Wei, Y. M. & Yang, H. A chlorine-tolerant nanofiltration membrane prepared by the mixed diamine monomers of PIP and BHTTM. *J. Membr. Sci.* **498**, 374-384 (2016).

43. Zhu, Y. Z. et al. Single-walled carbon nanotube film supported nanofiltration membrane with a nearly 10 nm thick polyamide selective layer for high-flux and high-rejection desalination. *Small* **12**, 5034-5041 (2016).

44. Wu, M. B. et al. Thin film composite membranes combining carbon nanotube intermediate layer and microfiltration support for high nanofiltration performances. *J. Membr. Sci.* **515**, 238-244 (2016).

45. Yang, X., Du, Y., Zhang, X., He, A. & Xu, Z. K. Nanofiltration membrane with a mussel-inspired interlayer for improved permeation performance. *Langmuir* **33**, 2318-2324 (2017).

46. Soyekwo, F. et al. Cellulose nanofiber intermediary to fabricate highly-permeable ultrathin nanofiltration membranes for fast water purification. *J. Membr. Sci.* **524**, 174-185 (2017).

47. Wang, J. J., Yang, H. C., Wu, M. B., Zhang, X. & Xu, Z. K. Nanofiltration membranes with cellulose nanocrystals as an interlayer for unprecedented performance. *J. Mater. Chem. A* **5**, 16289-16295 (2017).

48. Wang, Z. Y. et al. Nanoparticle-templated nanofiltration membranes for ultrahigh performance desalination. *Nat. Commun.* **9**, 2004 (2018).

49. Zhu, J. Y. et al. Rapid water transport through controllable, ultrathin polyamide nanofilms for high-performance nanofiltration. *J. Mater. Chem. A* **6**, 15701-15709 (2018).

50. Tan, Z., Chen, S. F., Peng, X. S., Zhang, L. & Gao, C. J. Polyamide membranes with nanoscale Turing structures for water purification. *Science* **360**, 518-521 (2018).
